# Loss of NR5A1 in mouse Sertoli cells after sex determination changes cellular identity and induces cell death by anoikis

**DOI:** 10.1242/dev.201710

**Published:** 2023-12-11

**Authors:** Sirine Souali-Crespo, Diana Condrea, Nadège Vernet, Betty Féret, Muriel Klopfenstein, Erwan Grandgirard, Violaine Alunni, Marie Cerciat, Matthieu Jung, Chloé Mayere, Serge Nef, Manuel Mark, Frédéric Chalmel, Norbert B. Ghyselinck

**Affiliations:** ^1^ Institut de Génétique et de Biologie Moléculaire et Cellulaire (IGBMC), Département de Génétique Fonctionnelle et Cancer, Centre National de la Recherche Scientifique (CNRS UMR7104), Institut National de la Santé et de la Recherche Médicale (INSERM U1258), Université de Strasbourg (UNISTRA), 1 rue Laurent Fries, BP-10142, F-67404 Illkirch Cedex, France; ^2^ Imaging Center, IGBMC, F-67404 Illkirch Cedex, France; ^3^ GenomEast Platform, France Génomique consortium, IGBMC, 1 rue Laurent Fries, F-67404 Illkirch Cedex, France; ^4^ Department of Genetic Medicine and Development, Faculty of Medicine, University of Geneva, CH-1211 Geneva 4, Switzerland; ^5^ Service de Biologie de la Reproduction, Hôpitaux Universitaires de Strasbourg (HUS), F-67000 Strasbourg, France; ^6^ Univ Rennes, EHESP, Inserm, Irset (Institut de recherche en santé, environnement et travail) - UMR_S 1085, F-35000 Rennes, France

**Keywords:** Gonad, Integrin, Leydig cell, Mutant, Testis, NR5A1/SF-1

## Abstract

To investigate the role of the nuclear receptor NR5A1 in the testis after sex determination, we analyzed mice lacking NR5A1 in Sertoli cells (SCs) from embryonic day (E) 13.5 onwards. Ablation of *Nr5a1* impaired the expression of genes characteristic of SC identity (e.g. *Sox9* and *Amh*), caused SC death from E14.5 onwards through a *Trp53*-independent mechanism related to anoikis, and induced disorganization of the testis cords. Together, these effects caused germ cells to enter meiosis and die. Single-cell RNA-sequencing experiments revealed that NR5A1-deficient SCs changed their molecular identity: some acquired a ‘pre-granulosa-like’ cell identity, whereas other reverted to a ‘supporting progenitor-like’ cell identity, most of them being ‘intersex’ because they expressed both testicular and ovarian genes. Fetal Leydig cells (LCs) did not display significant changes, indicating that SCs are not required beyond E14.5 for their emergence or maintenance. In contrast, adult LCs were absent from postnatal testes. In addition, adult mutant males displayed persistence of Müllerian duct derivatives, decreased anogenital distance and reduced penis length, which could be explained by the loss of AMH and testosterone synthesis due to SC failure.

## Teaser

**Summary:** Loss of NR5A1 after the sex determination period causes Sertoli cells to revert to supporting progenitor-like cells and transdifferentiate into pre-granulosa-like cells that die by a *Trp53*-independent mechanism.

## INTRODUCTION

In humans, disorders of sex development (DSDs) are caused by mutations in certain genes regulating gonad development. Crucially, these disorders provide an important system for understanding the molecular mechanisms that underpin cell differentiation (Eggers et al., 2014). Among the genes responsible for DSDs is *NR5A1* (Fabbri-Scallet et al., 2020), which encodes the orphan nuclear receptor NR5A1, a transcription factor that is expressed when cells acquire a fate of either adrenal or gonadal primordium (Morohashi et al., 2013). Consistent with a role in DSDs, NR5A1 has been reported to regulate proliferation, survival and differentiation of somatic progenitor cells through specific gene expression (Morohashi et al., 2020).

Both testes and ovaries originate from a bipotential gonad that contains the primordial germ cells (GCs) and early somatic progenitors. Around embryonic day (E) 11.5 in mice, it is these somatic progenitors that first undergo sex-specific cell differentiation into bipotential supporting progenitors (supPs), and then either into pre-Sertoli cells (SCs) in the testis upon SRY/SOX9 expression or into pre-granulosa (pGr) cells in the ovary upon WNT/CTNNB1 signaling stabilization (Nef et al., 2019). The sex-specific fate decision then propagates to GCs and to other somatic lineages in the testis, including the steroidogenic Leydig cells (LCs), which, in turn, drive the acquisition of primary and secondary sexual characteristics at later stages of development through hormone secretion (Yao et al., 2015; Rotgers et al., 2018; Nef et al., 2019).

Knockout of *Nr5a1* in mice results in agenesis of the adrenal gland and regression of the gonads by E11.5 due to apoptosis of somatic cells (Luo et al., 1994). From E11.5, NR5A1 and SRY are expressed in the bipotential supP cells of the male gonad, where they upregulate SOX9 expression, thereby initiating pre-SC and ultimately SC differentiation (Sekido and Lovell-Badge, 2008; Rotgers et al., 2018; Nef et al., 2019). Once specified, SCs start forming cords by enclosing GCs as early as E12.5 in mice (Cool et al., 2012), providing them with a specialized environment that promotes their survival and orchestrates their differentiation (Griswold, 2018). SCs also allow the differentiation of fetal LCs through paracrine desert hedgehog (DHH) signaling (Bitgood et al., 1996) and produce high levels of anti-Müllerian hormone (AMH) under the control of NR5A1 and SOX9, which triggers the regression of the Müllerian duct normally giving rise to female genitalia (Josso and Picard, 2022).

As gonads are absent in *Nr5a1*-null mice (Luo et al., 1994), studying the cell-specific role of NR5A1 in the developing testis required analysis of mice bearing tissue-targeted mutations. Here, we analyzed the outcome of *Nr5a1* ablation in SCs from E13.5 onwards. We show that loss of NR5A1 from this stage caused some SCs to revert to a supP cell-like state, other SCs to sexually transdifferentiate into female somatic pGr cells, and all of them to die by a *Trp53*-independent mechanism related to anoikis.

## RESULTS

### Deletion of *Nr5a1* in SCs

**Fig. 1.**
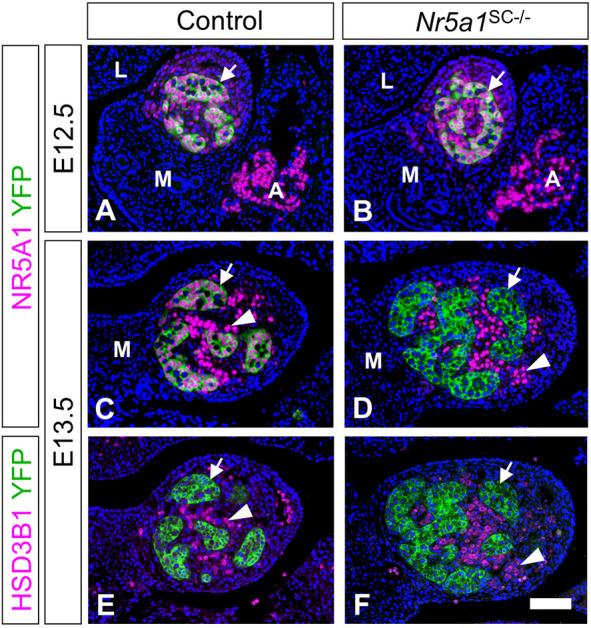
**Ablation of *Nr5a1* in SCs is efficient from E13.5 onwards.** (A-F) Detection of NR5A1 (magenta nuclear signals), YFP (green cytoplasmic signals) and HSD3B1 (magenta cytoplasmic signals) by IHC on histological sections of control (A,C,E) and *Nr5a1*^SC−/−^ (B,D,F) testes at E12.5 (A,B) and E13.5 (C-F). Efficient excision of the reporter transgene by Cre was assessed by YFP expression in Sertoli cells (SCs) (arrows) at E12.5 (A,B). However, loss of NR5A1 in SCs was only achieved at E13.5 (compare C with D). At this stage, expression of NR5A1 was maintained in Leydig cells (LCs) (arrowheads in C,D), as identified on consecutive sections by their expression of HSD3B1 (E,F). Nuclei were counterstained with DAPI (blue signal). Images are representative of three experiments. ‘A’, adrenal primordium; ‘L’, liver; ‘M’, mesonephros. Scale bar: 50 µm (A-F).

To achieve *Nr5a1* ablation in SCs after sex determination, we introduced the *Plekha5*^Tg(AMH-cre)1Flor^ transgene (Lécureuil et al., 2002) in mice bearing *loxP*-flanked alleles of *Nr5a1* and a Cre-dependent yellow fluorescent protein (YFP) reporter transgene (Srinivas et al., 2001). Cre-mediated recombination was assessed by immunohistochemistry (IHC) using antibodies recognizing YFP. Surprisingly, YFP was detected in all SCs of E12.5 gonads (arrows, Fig. 1A,B), indicating that Cre-mediated excision occurred earlier than anticipated (Lécureuil et al., 2002). However, the NR5A1 protein was still detected in SCs at E12.5 (Fig. 1A,B). It was lost in mutant testes at E13.5 (Fig. 1D) but maintained in control testes (Fig. 1C). Importantly, NR5A1 was still detected in the nuclei of LCs, identified by their expression of 3-β-hydroxysteroid dehydrogenase type 1 (HSD3B1), in both control and mutant testes (Fig. 1E,F). Thus, NR5A1 was lost selectively in SCs from E13.5 onwards, generating males hereafter referred to as *Nr5a1*^SC−/−^ mutants.

### Ablation of *Nr5a1* in SCs impairs expression of SOX9, SOX8, SOX10 and AMH

**Fig. 2.**
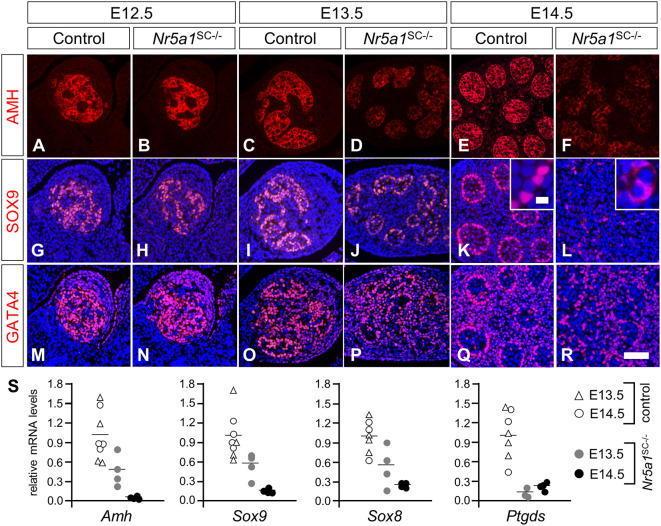
**Ablation of *Nr5a1* in SCs impairs AMH and SOX9 expression.** (A-R) Detection of AMH, SOX9 and GATA4 (red signals) on transverse histological sections of control (A,C,E,G,I,K,M,O,Q) and *Nr5a1*^SC−/−^ (B,D,F,H,J,L,N,P,R) testes at E12.5 (A,B,G,H,M,N), E13.5 (C,D,I,J,O,P) and E14.5 (E,F,K,L,Q,R). Nuclei were counterstained with DAPI (blue signals in G-R). Insets (K,L) are high magnifications showing nuclear localization of SOX9 in control SCs versus cytoplasmic localization in mutant SCs. Note that at each developmental stage, detection of AMH, SOX9 and GATA4 was performed on consecutive sections. Images are representative of three experiments. Scale bars: 50 µm (A-R); 5 μm (insets in K,L). (S) RT-qPCR analyses comparing the expression levels of *Amh*, *Sox9*, *Sox8* and *Ptgds* mRNAs in whole-testis RNA extracted from control (*n*=4) and *Nr5a1*^SC−/−^ (*n*=4) testes at E13.5 and E14.5 as indicated. Each point represents the mean value of a technical triplicate, and the bars indicate the mean values.

As NR5A1 regulates the expression of *Amh* and *Sox9* genes (De Santa Barbara et al., 1998; Sekido and Lovell-Badge, 2008), we tested the expression of the respective proteins by IHC. Both AMH and SOX9 were detected at normal levels at E12.5 (Fig. 2A,B,G,H). Their expression decreased progressively between E13.5 and E14.5 in *Nr5a1*^SC−/−^ mutant testes (Fig. 2C-F,I-L). The expression of SOX8 and SOX10 was also decreased (Fig. S1). Accordingly, the steady state levels of *Amh*, *Sox9* and *Sox8* mRNAs were decreased in *Nr5a1*^SC−/−^ testes (Fig. 2S). The mRNA levels of prostaglandin D2 synthase (*Ptgds*), a SOX9 target gene (Wilhelm et al., 2007), were also reduced in *Nr5a1*^SC−/−^ testes. This finding may explain the cytoplasmic instead of the nuclear localization of SOX9 in some mutant SCs (compare insets, Fig. 2K,L) because prostaglandin D2, the end product of PTGDS, is required for SOX9 nuclear translocation (Malki et al., 2005).

In parallel, we analyzed the expression of GATA4 and WT1 transcription factors, which are important for *Amh* expression and SC differentiation (Tremblay and Viger, 1999; Buganim et al., 2012). Both were detected at similar levels in SC nuclei of control and mutant testes, from E12.5 to E14.5 (Fig. 2M-R; Fig. S1).

### Ablation of *Nr5a1* in SCs induces disorganization of testis cords

**Fig. 3.**
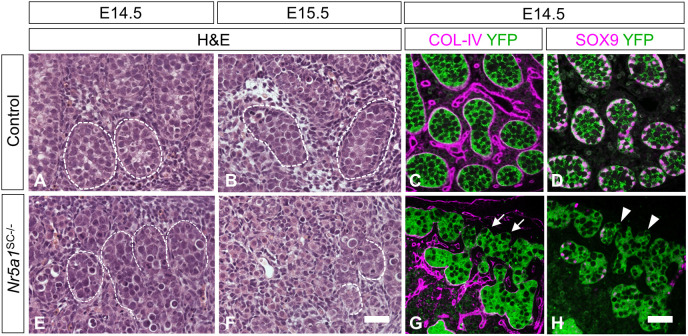
**Ablation of *Nr5a1* in SCs induces testis cord disorganization.** (A,B,E,F) Histological sections through E14.5 (A,E) and E15.5 (B,F) control (A,B) and *Nr5a1*^SC−/−^ (E,F) testes stained with Hematoxylin and Eosin (H&E). In controls, the seminiferous cords were well defined (closed dotted lines), whereas in mutants, they were poorly defined (open dotted lines). (C,D,G,H) Detection of COL-IV, SOX9 (magenta signals) and YFP (green signal) on transverse histological sections of control (C,D) and *Nr5a1*^SC−/−^ (G,H) testes at E14.5. Note that C,D and G,H are consecutive sections. Arrows (G) point to the loss of COL-IV at the periphery of seminiferous cords, where SOX9 is lost in SCs (arrowheads in H). Images are representative of three experiments. Scale bars: 10 µm (A,B,E,F); 15 µm (C,D,G,H).

In histological sections at E14.5, the majority of *Nr5a1*^SC−/−^ seminiferous cords displayed reduced diameters, as well as poorly defined and discontinuous contours (Fig. 3E). At E15.5, the cords had almost completely disappeared (Fig. 3F). Such irregularities were never observed in the control testes (Fig. 3A,B).

To visualize seminiferous cord basement membranes, we analyzed the expression of collagen type IV (COL-IV) using an antibody against COL4A1 to COL4A5 by IHC. At E14.5, COL-IV surrounded the entire periphery of all cords in control testes (white border, Fig. 3C). In *Nr5a1*^SC−/−^ testes, COL-IV was greatly reduced and even absent from the periphery of many cords (arrows, Fig. 3G) and SOX9 was expressed in all SCs (Fig. 3D), in areas corresponding to regions where SOX9 expression was also lost (arrowheads, Fig. 3H). As *Col4a1* and *Col4a2* are regulated by SOX9 (Sumi et al., 2007), it is proposed that loss of SOX9 in NR5A1-deficient SCs resulted in loss of COL-IV and altered the basement membrane, which, in turn, disorganized the cord structure. Knowing that testis cord formation and vasculature are closely linked (Brennan et al., 2003), the question arose whether the vasculature was affected in *Nr5a1*^SC−/−^ testes. We show that it was normal, at least until E14.5 (Fig. S2).

### NR5A1-deficient SCs die through a TRP53-independent mechanism

**Fig. 4.**
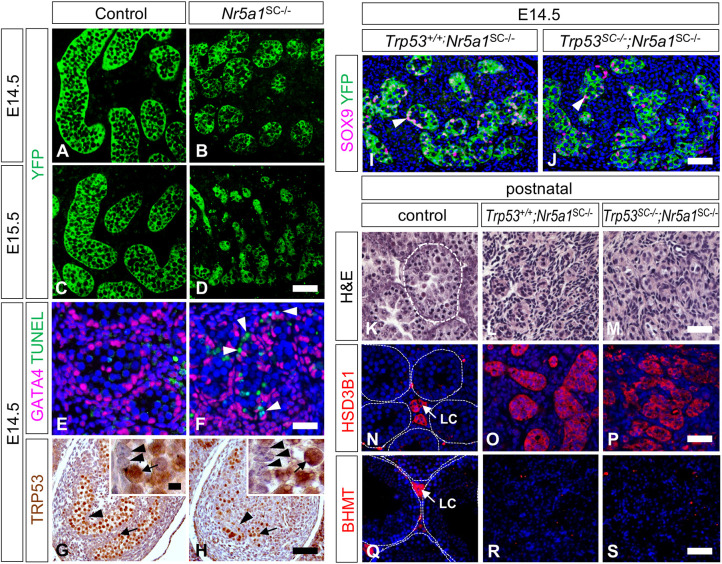
**NR5A1-deficient SCs die even when they lack TRP53.** (A-D) Detection of YFP (green signal) on histological sections from control (A,C) and *Nr5a1*^SC−/−^ (B,D) testes at E14.5 (A,B) and E15.5 (C,D). (E,F) Detection of GATA4 (magenta signal) and TUNEL-positive cells (green signal) on histological sections of control (E) and *Nr5a1*^SC−/−^ (F) testes at E14.5. Nuclei were counterstained with DAPI (blue signal). Arrowheads point to TUNEL-positive SCs in the mutant. (G,H) Detection of TRP53 (brown signal) on histological sections of control (G) and *Nr5a1*^SC−/−^ (H) testes at E14.5. Counterstaining was with H&E. The insets show higher magnifications. Arrowheads and arrows point to SCs and GCs, respectively. (I,J) Detection of SOX9 (magenta signal) and YFP (green signal) on histological sections of *Trp53*^+/+^;*Nr5a1*^SC−/−^ (I) and *Trp53*^SC−/−^;*Nr5a1*^SC−/−^ (J) testes at E14.5. Arrowheads point to SCs. (K-M) H&E staining of histological sections from control (K), *Trp53*^+/+^;*Nr5a1*^SC−/−^ (L) and *Trp53*^SC−/−^;*Nr5a1*^SC−/−^ (M) testes at postnatal day (PND) 15. (N-S) Detection of HSB3B1 (N-P) and BHMT (Q-S) (red signals) on histological sections of control (N,Q), *Trp53*^+/+^;*Nr5a1*^SC−/−^ (O,R) and *Trp53*^SC−/−^;*Nr5a1*^SC−/−^ (P,S) testes at PND15 (N-P) and PND60 (Q-S). Nuclei were counterstained with DAPI (blue signal). The dotted white lines (in K,N,Q) delineate seminiferous tubules. Images are representative of three experiments. LC, Leydig cell. Scale bars: 25 µm (A-D,I,J); 15 µm (E,F); 100 µm (G,H); 80 µm (K-P); 20 µm (Q-S).

By YFP expression, we quantified the surface area occupied by SCs (green pixels) relative to the whole testis surface area (Fig. 4A-D). At E14.5, this percentage was about 30% lower in mutant than control testes [28.3±2.0% (*n*=5) versus 37.9±1.4% (*n*=5), respectively; indicated as mean±s.e.m., *P*<0.01, bilateral, unpaired, Student's *t*-test]. This suggested that NR5A1-deficient SCs had either silenced expression of YFP or had disappeared from the testis. If YFP expression was silenced, the Cre-recombined *Nr5a1* allele (L–) should have been present in the testes at birth. It should no longer be detected if SCs were lost. In fact, PCR analysis of genomic DNA showed that the L− allele was not present in *Nr5a1*^SC−/−^ mutants (Fig. S3A), indicating that NR5A1-deficient SCs were actually lost from the mutant testis. Consistent with mutant SC elimination, numerous cells displaying features of SCs (i.e. located at the periphery of the testis cords, amongst GATA4-expressing SCs) showed positive staining by terminal deoxynucleotidyl transferase dUTP nick end labeling (TUNEL) assays in E14.5 *Nr5a1*^SC−/−^ testes (arrowheads, Fig. 4E,F). This indicates that NR5A1-deficient SCs died progressively.

Previously, ablation of *Nr5a1* in SCs was proposed to increase phosphorylation of TRP53 and to induce apoptosis (Anamthathmakula et al., 2019). However, TRP53 could not be detected by IHC in SCs at E14.5 (Fig. 4G,H). Therefore, to test whether TRP53 was involved in SC death, we set up a functional assay. We introduced conditional alleles of *Trp53* (Jonkers et al., 2001) into *Nr5a1*^SC−/−^ mutants carrying the YFP reporter transgene. Efficient ablation of *Trp53* was assessed by PCR on genomic DNA extracted from YFP-positive SCs purified by fluorescence-activated cell sorting (FACS) (Fig. S3B). Contrary to the expectations, both *Nr5a1*^SC−/−^ and *Nr5a1*^SC−/−^;*Trp53*^SC−/−^ mutant testes at E14.5 displayed identical defects, including the loss of SOX9 expression and decreased number of SCs (Fig. 4I-J, compare with Fig. 3D). In addition, both *Nr5a1*^SC−/−^ and *Nr5a1*^SC−/−^;*Trp53*^SC−/−^ mutant testes lacked SC-surrounded seminiferous tubules at postnatal day (PND) 15. Instead, they contained interstitial cells, including HSD3B1-positive fetal LCs (Fig. 4K-P). This indicates that TRP53 did not play a role in the death of NR5A1-deficient SCs. In adult *Nr5a1*^SC−/−^ testes none of the interstitial of cells expressed the adult LC-specific marker BHMT (Sararols et al., 2021) suggesting that this cell type was unable to emerge during postnatal testis development (Fig. 4Q-S). Altogether, these data indicate that NR5A1-deficient SCs died in a TUNEL-positive but TRP53-independent manner, resulting in postnatal testes containing fetal but not adult LCs.

### *Nr5a1*^SC−/−^ adult males exhibit retention of Müllerian duct derivatives

**Fig. 5.**
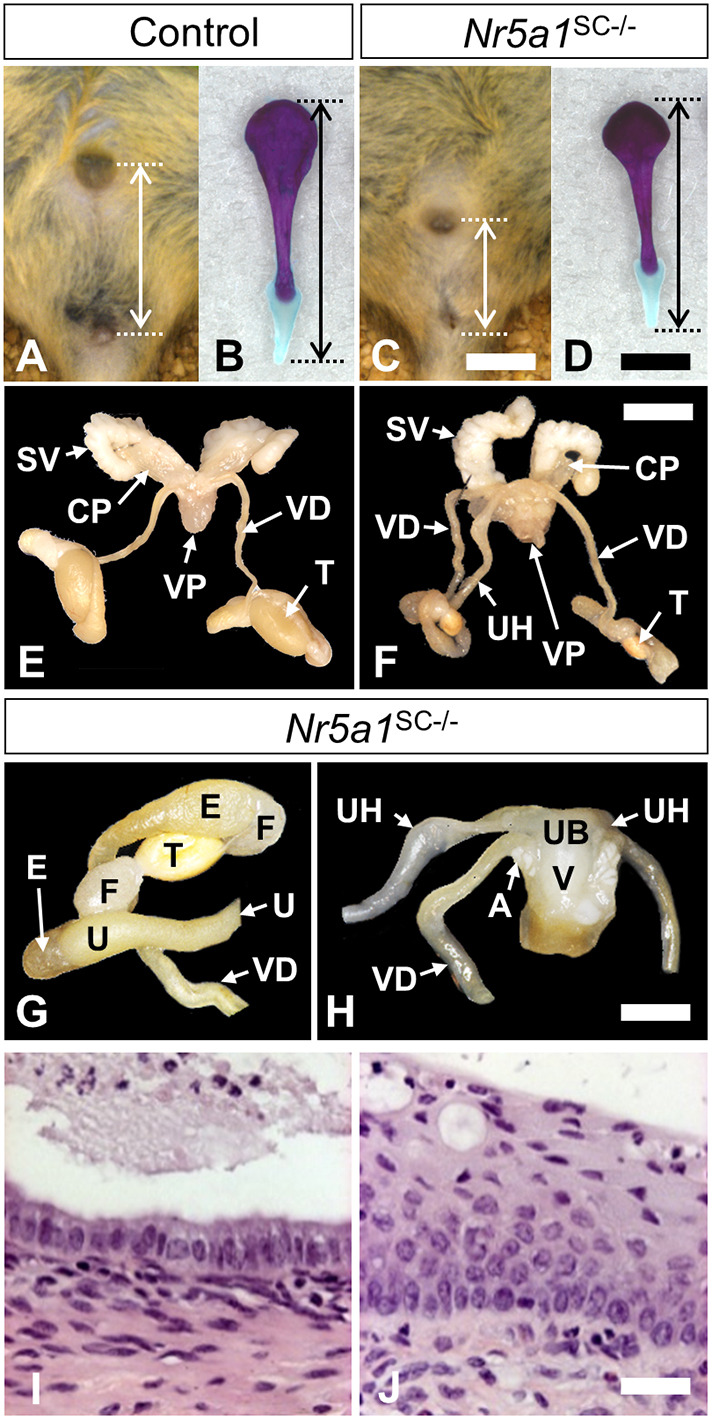
**Abnormal external genitalia and retention of Müllerian duct derivatives in *Nr5a1*^SC−/−^ males.** (A,C) Anogenital distance in control (A) and *Nr5a1*^SC−/−^ (B) males at PND60. (B,D) Alizarin Red- and Alcian Blue-stained penis bones from control (A) and *Nr5a1*^SC−/−^ (B) males at PND60. (E-H) Reproductive tracts of control (E) and *Nr5a1*^SC−/−^ (F-H) males at PND60. (I-J) Histological sections through the *Nr5a1*^SC−/−^ genital tract shown in H and stained with H&E. The uterine horn displays a simple columnar epithelium (I) and the vaginal epithelium is typically stratified and squamous (J). Images are representative of three experiments. A, ampullary gland; CP, cranial prostate; E, epididymis; F, fat pad; SV, seminal vesicle; T, testis; U, uterus, UB, uterus body; UH, uterine horn; V, vagina; VD, vas deferens; VP, ventral prostate. Scale bars: 4 mm (A,C); 2 mm (B,D); 10 mm (E,F); 5 mm (G,H) 10 µm (I,J).

We analyzed the outcome of the loss of SCs and AMH production in *Nr5a1*^SC−/−^ mutants at PND60. The males were all sterile and had a shorter anogenital distance (AGD) index [0.30±0.01 mm/g of body weight in mutants (*n*=16) versus 0.36±0.01 mm/g in controls (*n*=17); indicated as mean±s.e.m.; *P*<0.001, bilateral, unpaired, Student's *t*-test] (Fig. 5A,C). At autopsy (*n*=8), they displayed normal derivatives of the Wolffian ducts (e.g. epididymis, vas deferens and seminal vesicle) and the urogenital sinus (i.e. prostatic lobes) (Fig. 5E,F). However, they also displayed Müllerian duct derivatives (Fig. 5F-H), namely, a vagina, a uterine body and bilateral uterine horns, which were either incomplete and truncated (six out of eight), or complete on one side (two out of eight). Histological analyses revealed that the uterine body and vagina displayed a columnar epithelium (Fig. 5I) and a stratified squamous epithelium (Fig. 5J), as anticipated for these female organs.

In addition, *Nr5a1*^SC−/−^ males displayed 40% lighter seminal vesicles [4.71±0.32 mg/g of body weight in mutants (*n*=17) versus 7.43±0.63 mg/g in controls (*n*=16); indicated as mean±s.e.m.; *P*<0.05, bilateral, unpaired, Student's *t*-test] and 10% shorter penis bones [6.3±0.2 mm in mutants (*n*=17) versus 7.1±0.2 mm in controls (*n*=16); indicated as mean±s.e.m.; *P*<0.05, bilateral, unpaired, Student's *t*-test] (Fig. 5B,D). As AGD, seminal vesicle growth and penis bone length vary as a function of androgen exposure (Welsh et al., 2014), we tested blood testosterone levels. They were comparable at birth [0.25±0.06 ng/ml in mutants (*n*=10) versus 0.22±0.03 ng/ml in controls (*n*=10); indicated as mean±s.e.m.; *P*=0.59, bilateral, unpaired, Student's *t*-test], and at PND60 [0.38±0.06 ng/ml in controls (*n*=17) versus 0.43±0.10 ng/ml in controls (*n*=16); indicated as mean±s.e.m.; *P*=0.68, bilateral, unpaired, Student's *t*-test], indicating normal testosterone production.

### Transcriptomic signatures of cells in control and NR5A1-deficient testes

**Fig. 6.**
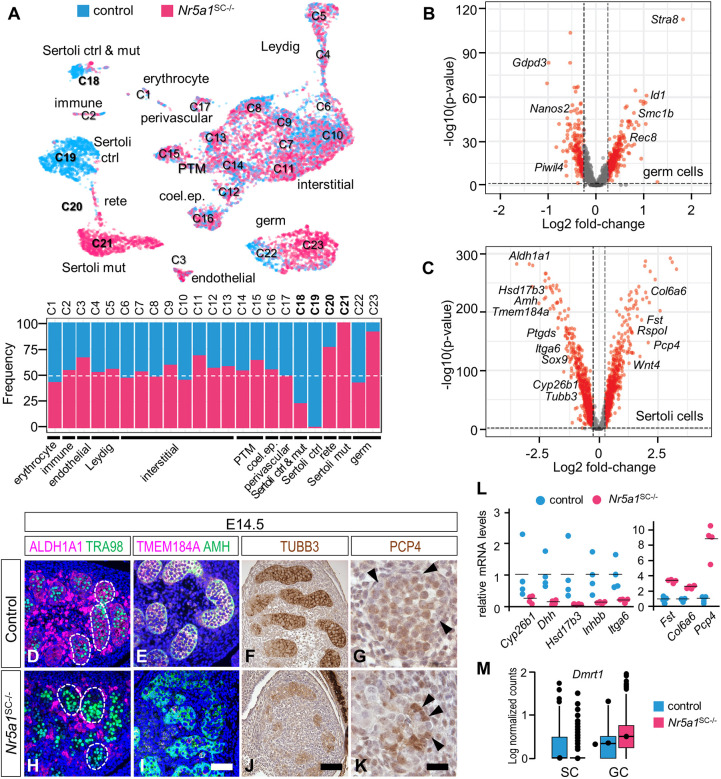
**Transcriptomes of control and *Nr5a1*^SC−/−^ testicular cells at a single-cell resolution.** (A) Uniform manifold approximation and projection (UMAP) plot of single-cell transcriptomes from control (blue) and *Nr5a1*^SC−/−^ (pink) testes at E14.5, partitioned into 23 cell clusters (named C1-C23) using Seurat graph-based clustering. The proportion of control and mutant cells in each cluster is indicated below as colored bars. coel. ep., coelomic epithelium; ctrl, control; mut, mutant; PTM, peritubular myoid cell. (B,C) Volcano plots of differential gene expression in GCs (panel B, clusters C22 and C23) and SCs (panel C, clusters C18, C19 and C21) from control and *Nr5a1*^SC−/−^ testes. Red dots correspond to genes dysregulated more than 1.2-fold. (D-K) Detection of ALDH1A1, TMEM184A (magenta signals), GCNA (recognized by the TRA98 antibody), AMH (green signals), TUBB3 and PCP4 (brown signals) on transverse histological sections of control (D-G) and *Nr5a1*^SC−/−^ (H-K) testes at E14.5. Nuclei were counterstained with DAPI (blue signal in D,E,H,I) or with H&E (F,G,J,K). The dotted lines in D,H delimit seminiferous cords. The arrowheads in G,K point to SC nuclei. Images are representative of three experiments. (L) RT-qPCR analyses comparing the expression levels of selected genes using RNA extracted from control (*n*=4) and *Nr5a1*^SC−/−^ (*n*=4) whole testes at E14.5. Each point represents the mean value of a technical triplicate, and the bars indicate the mean values. (M) Tukey box plots illustrating medians, ranges and variabilities of log-normalized expression of *Dmrt1* in SCs and GCs. Scale bars: 25 µm (D,E,H,I); 100 µm (F,J); 15 µm (G,K).

We then performed single-cell RNA-sequencing (scRNA-seq) experiments using dissociated cell suspensions obtained from 12 control and 16 *Nr5a1*^SC−/−^ whole testes at E13.5 and E14.5. After data processing and quality control, we assembled an atlas composed of 3988 control and 5010 mutant testicular cells. On average, we detected 13,860 unique molecular indices (UMIs) and approximately 3500 genes for each cell. The 8998 testicular cells were partitioned into 23 cell clusters (termed C1-C23) and projected into a two-dimensional space (Fig. 6A; Table S1). Known marker genes of distinct cell types were used to identify each cluster (Fig. S4).

Very few changes in gene expression were observed between control and *Nr5a1*^SC−/−^ testes for endothelial, immune and perivascular cells. In LCs, interstitial cells, and coelomic epithelium and peritubular myoid (PTM) cells, the expression of 59, 49 and 47 genes, respectively, was significantly (*P*<0.05) dysregulated (Table S2). Functional analysis revealed that downregulated genes were related to ribosome biosynthesis in LCs and interstitial cells, and epithelium development in coelomic epithelium and PTM cells (Fig. S5; Table S3). In GCs, the expression of 636 genes was significantly dysregulated (Table S2); among these, we found that *Stra8* and *Rec8* were upregulated, whereas *Nanos2* and *Piwil4* were downregulated (Fig. 6B). Processes such as DNA recombination, meiotic cell cycle and oocyte differentiation were identified among the Gene Ontology (GO) terms associated with upregulated genes (Fig. S5; Table S3). Accordingly, we showed that GCs in *Nr5a1*^SC−/−^ testes at E14.5 and E15.5 were in the S phase of meiotic prophase, instead of becoming mitotically quiescent, as in the control situation (Fig. S6A-G). In addition, many GCs of *Nr5a1*^SC−/−^ testes expressed meiotic proteins, such as STRA8, REC8 and H2AFX (encoded by *H2ax*) (Fig. S6H-O), and died (Fig. S6P-R).

Three clusters (C18, C19 and C21) were identified as SCs. The C18 cluster included both control and NR5A1-deficient SCs (Fig. 6A) and had hallmarks of mitotic cells (Fig. S4B). The C19 and C21 clusters consisted almost exclusively of control and NR5A1-deficient SCs, respectively (Fig. 6A). In total, 1671 genes were significantly dysregulated in SCs lacking NR5A1 (Table S2), among which *Amh*, *Ptgds* and *Sox9* were downregulated as anticipated (see above), whereas *Fst*, *Rspo1* and *Wnt4* were upregulated (Fig. 6C). The expression of *Dmrt1* was reduced in SCs and increased in GCs of *Nr5a1*^SC−/−^ testes (Fig. 6M). We confirmed the dysregulated expression of some genes by IHC (Fig. 6D-K) and reverse-transcription quantitative PCR (RT-qPCR) (Fig. 6L), thereby validating the scRNA-seq experiment. Based on the fact that rete testis cells express *Aldh1a3*, *Pax8* and *Nr5a1* (Mayère et al., 2022), cluster C20 was assigned the rete testis identity (Fig. S4). The cells in this cluster were organized as a continuum, starting from C19 with true SCs (*Nr5a1*^+^, *Yfp*^−^, *Pax8*^−^ and *Aldh1a3*^−^) and extending with rete cells (*Nr5a1*^+^, *Yfp*^−^, *Pax8*^+^ and *Aldh1a3*^+^) derived from both control and *Nr5a1*^SC−/−^ testes. Accordingly, IHC experiments showed that rete testis cells actually expressed ALDH1A3, PAX8 and NR5A1, but not YFP, in both control and *Nr5a1*^SC−/−^ testes (Fig. S7). This can be explained by the fact that the *Plekha5*^Tg(AMH-cre)1Flor^ transgene is not active in the rete testis cells (Teletin et al., 2019).

### Death of NR5A1-deficient SCs is associated with anoikis

**Fig. 7.**
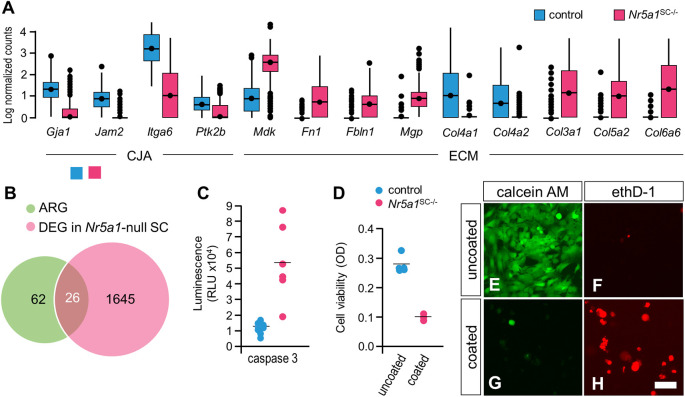
**NR5A1-deficient SCs die by anoikis.** (A) Tukey box plots illustrating medians, ranges and variabilities of log-normalized expression of the selected genes involved in either cell junction/adhesion (CJA) or ECM composition. (B) Venn diagram showing overlap between anoikis-related genes (ARG) and DEGs in NR5A1-deficient SCs. (C) Caspase 3 activity measured in FACS-purified control (*n*=8 batches) and NR5A1-deficient SCs (*n*=6 batches). RLU, relative luminescence units. (D) Cell viability assay using MSC-1 SCs plated in control (uncoated, *n*=5 wells) or anchorage-resistant (coated, *n*=3 wells) plates. Survival of SCs was severely compromised when they could not attach to the plate. Each point in C,D represents the value measured for individual batches of SCs or individual wells, and the bars indicate the mean values. (E-H) Representative images of anoikis assay using MSC-1 SCs seeded on uncoated (E,F) or anchorage-resistant (coated, G,H) plates. Live cells (green signal) were detected with calcein AM (E,F), whereas dead or dying cells (red signal) were detected with ethD-1 (G,H). The MSC-1 SCs could grow in the control (uncoated) plate, but died in the anchorage-resistant (coated) plate. Images are representative of three experiments. Scale bars: 15 µm (E-H).

Functional analysis of the differentially expressed genes (DEGs) between control and NR5A1-deficient SCs highlighted GO terms such as ‘extracellular matrix’ (ECM), ‘basement membrane’, ‘cell-substrate adhesion’ and ‘integrin binding’ (Fig. S5; Table S3). Accordingly, the expression of genes involved in cell junction/adhesion (e.g. *Gja1*, *Jam2* and *Ptk2b*) and integrin signaling (e.g. *Itga6*) was reduced in mutant SCs. As for ECM component genes, some were downregulated (e.g. *Col4a1* and *Col4a2*), whereas others were upregulated (e.g. *Col3a1*, *Col5a2*, *Col6a6*, *Fn1*, *Fbln1*, *Mdk* and *Mgp*) in mutant SCs (Fig. 7A). Upon further searching for ligand-receptor interactions in our dataset, we identified a dysregulated network related to integrin-dependent cell adhesion (Fig. S8). These findings indicate that NR5A1-deficient SCs lost their appropriate cell-cell and cell-ECM integrin-dependent interactions, and are consistent with the basement membrane alteration described above (see Fig. 3). This raised the possibility that NR5A1-deficient SCs die by detachment-induced cell-death (also known as anoikis).

Anoikis is the induction of apoptosis in anchorage-dependent cells and it occurs upon loss of attachment to the ECM (Gilmore, 2005; Vachon, 2011). Indeed, functional analysis of overexpressed genes in NR5A1-deficient SCs revealed ‘regulation of cell death’ and ‘regulation of apoptotic process’ among the altered functions (Fig. S5; Table S3). Furthermore, 26 of the 88 anoikis-related genes with a GeneCards score greater than 2.0 were deregulated in NR5A1-deficient SCs (Fig. 7B; Table S4). Similar to apoptosis, anoikis leads to the activation of caspases, through either the intrinsic or extrinsic apoptotic pathway. Therefore, the quantification of caspase 3 activity can serve as a marker of the anoikis process (Tedja et al., 2021). Using FACS-purified, YFP-positive SCs at E13.5, we found that caspase 3 activity was indeed 4-fold higher in NR5A1-deficient SCs than in control SCs (Fig. 7C). In addition, using the mouse SC line MSC-1 as a surrogate model of control SCs (McGuinness et al., 1994), we showed that SCs survived when cultured *in vitro* on normal (uncoated) plates, but died when cultured on anchorage-resistant (hydrogel-coated) plates (Fig. 7D-H). Taken together, these results indicate that SCs normally rely on cell-ECM attachment to survive and that this property is compromised when NR5A1 is lost.

### *Nr5a1* ablation alters the molecular identity of SCs

**Fig. 8.**
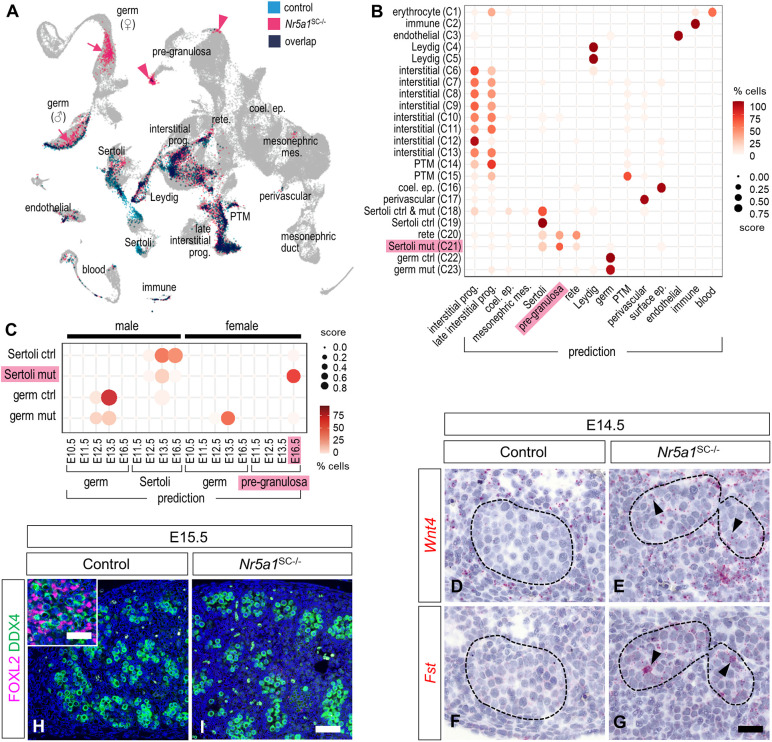
**Ablation of *Nr5a1* induces some SCs to acquire a pre-granulosa-like cell identity.** (A) Predicted projection of control (blue) and *Nr5a1*^SC−/−^ (pink) cells on a reference single-cell transcriptomic atlas of gonad development. Pink arrows and arrowheads point to the locations where GCs and SCs of the *Nr5a1*^SC−/−^ testes matched best, respectively. (B,C) Dot plots representing the predicted association of cell clusters from the control and *Nr5a1*^SC−/−^ single-cell dataset (*y*-axes) to cell types (B) and to sex and developmental stages (C) according to the atlas (*x*-axes). The dot size represents the prediction score (ranging from 0.0 to 1.0) of a given cell cluster to be associated with a given cell type based on the TransferData function implemented in Seurat. The color intensity (from white to dark red) indicates the percentage of cells of a given cell cluster that have been associated with a given cell type. Legend: coel. ep., coelomic epithelium; ctrl, control; ep., epithelium; mes., mesenchyme; mut, mutant; prog., progenitor; PTM, peritubular myoid cell. (D-G) *In situ* hybridization showing expression of *Wnt4* (D,E) and *Fst* (F,G) mRNAs detected by red color staining in control (D,F) and *Nr5a1*^SC−/−^ (E,G) testes at E14.5. Almost no dots were observed in the seminiferous cords of the control testes. In contrast, red dots indicative of *Wnt4* or *Fst* mRNA were present inside the seminiferous cords in *Nr5a1*^SC−/−^ testis (arrowheads). The seminiferous cords are delineated by dotted lines. (H,I) Detection of FOXL2 (magenta signal) and DDX4 (green signal) on transverse histological sections of control (H) and *Nr5a1*^SC−/−^ (I) testes at E15.5. Nuclei were counterstained with DAPI (blue signal). The inset in H shows detection of FOXL2 in an E15.5 fetal ovary, used as a positive control for IHC. Images are representative of three experiments. Scale bars: 15 µm (D-G); 25 µm (H,I) 50 µm (H, inset).

The NR5A1-deficient SCs in cluster C21 gained expression of *Wnt4*, *Fst* and *Rspo1* (Fig. 6C; Table S2), which are hallmarks of fetal ovarian somatic cells (Eggers et al., 2014). This raised the question as to whether some SCs had adopted a sex-reversed cell differentiation pathway. To test for this possibility, we compared our data with a reference atlas of embryonic and fetal gonadal cell transcriptomes ranging from E10.5 to E16.5 (Mayère et al., 2022). After processing this atlas with the pipeline that we used for our dataset (Fig. S9), we mapped our dataset to the reference atlas (Fig. 8A). Most control and *Nr5a1*^SC−/−^ cell clusters were predicted to correspond to clusters with identical or similar identities (Fig. 8B). Consistent with our expectation, cluster C20 was predicted to correspond best to rete testis cells (see above, Fig. S7). In addition, the majority of GCs from control testes matched with E13.5 male GCs in the atlas, whereas a large proportion of those from *Nr5a1*^SC−/−^ testes matched with E13.5 female (i.e. meiotic) GCs (Fig. 8C) as shown by IHC analyses (Fig. S6), thus validating the strategy in general. Most importantly, NR5A1-deficient SCs (cluster C21) corresponded not only to E13.5 SCs, but also to E16.5 pGr cells in the atlas (Fig. 8B,C). Thus, the identity of a fraction of NR5A1-deficient SCs was lost and they acquired a pGr-like cell identity. Accordingly, GO term analysis assigned upregulated genes in NR5A1-deficient SCs to the WNT signaling pathway, which is known to drive pGr cell differentiation in the fetal ovary (Chassot et al., 2014) (Fig. S5; Table S3), and we observed expression of *Wnt4* and *Fst* mRNA in the seminiferous cords of *Nr5a1*^SC−/−^ but not control testes (Fig. 8D-G). However, the process of sexual transdifferentiation remained abnormal, as IHC experiments showed no evidence of FOXL2 expression in the *Nr5a1*^SC−/−^ mutant testes at E15.5 (Fig. 8H,I).

**Fig. 9.**
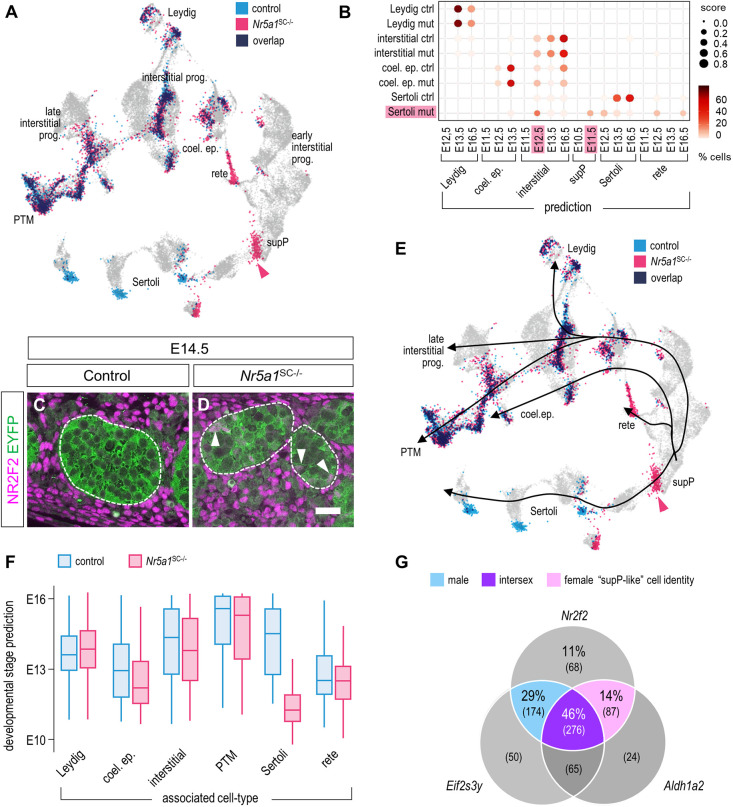
**Ablation of *Nr5a1* induces some SCs to acquire a supP-like cell identity.** (A) Predicted projection of control (blue) and *Nr5a1*^SC−/−^ (pink) somatic cells on a reference single-cell transcriptomic atlas of somatic cell development. (B) Dot plots representing the predicted association of cell clusters from the control and *Nr5a1*^SC−/−^ single-cell dataset (*y*-axis) to cell types and developmental stages according to the atlas (*x*-axis). The dot size represents the prediction score (ranging from 0.0 to 1.0) of a given cell cluster to be associated with a given cell type based on the TransferData function implemented in Seurat. The color intensity (from white to dark red) indicates the percentage of cells of a given cell cluster that have been associated with a given cell type. coel. ep., coelomic epithelium; ctrl, control; mut, mutant; prog., progenitor; PTM, peritubular myoid; supP, supporting progenitor. (C,D) Detection of NR2F2 (magenta signal) and YFP (green signal) on transverse histological sections of control (C) and *Nr5a1*^SC−/−^ (D) testes at E14.5. The arrowheads point to NR5A1-deficient (YFP-positive) SCs expressing NR2F2 (pink nuclei). Images are representative of three experiments. Scale bar: 15 µm. (E) Trajectories inferred over the developmental stages for the major somatic cell types of the testis superimposed to the predicted projection as in panel A. (F) Box plot showing the developmental stage prediction for each somatic cell type of the male control (blue boxes) and *Nr5a1*^SC−/−^ (pink boxes) testes. The population of NR5A1-deficient SCs appeared younger (ranging from E10 to E12) than the control SC population (ranging from E12 to E16). (G) Venn diagram showing the percentages of cells expressing *Nr2f2*, *Eif2s3y* and *Aldh1a2*, alone or in combination. Cells expressing *Nr2f2* and *Eif2s3y* were assigned a male supP-like cell identity (blue color), cells expressing *Nr2f2* and *Aldh1a2* were assigned a female supP-like cell identity (pink color) and cells expressing *Nr2f2*, *Eif2s3y* and *Aldh1a2* were assigned an intersex supP-like identity (purple color).

To test whether some SCs had adopted a cellular identity corresponding to that of another cell type, we then mapped the somatic cells of our dataset (Fig. S10) to the male somatic cells of the reference atlas (Fig. S11; Mayère et al., 2022). Again, control and most of *Nr5a1*^SC−/−^ cells matched to cell types with the expected identities (Fig. 9A), with the notable exception of NR5A1-deficient SCs, which matched to cell types such as E12.5 interstitial and E11.5 supP cells, in addition to SCs and rete testis cells (Fig. 9B). This raised the possibility that some mutant SCs reverted to somatic cells of an earlier developmental stage. Consistent with this idea, NR5A1-deficient SCs expressed *Nr2f2* mRNA (arrowhead, Fig. S12A), and the NR2F2 protein could be detected in some SCs of *Nr5a1*^SC−/−^ but not control testes (Fig. 9C,D). Interestingly, NR2F2 is a hallmark of bipotential supP cells (Stévant et al., 2019). To test whether NR5A1-deficient SCs indeed reverted to ‘supP-like’ cells, we inferred cell differentiation lineages and pseudo-time using the Slingshot method (Street et al., 2018). We obtained linear trajectories for each cell type, along which the majority of NR5A1-deficient SCs were at the starting point of the SC differentiation pathway, where supP cells are located (arrowhead, Fig. 9E). Correspondingly, prediction of developmental stage and pseudo-time revealed that NR5A1-deficient SCs matched to the E10-E12 stages, whereas control SCs matched to the E13-E15 stages (Fig. 9F; Fig. S12B). This supported the idea that loss of *Nr5a1* shifts some SCs to an earlier, supP-like state of differentiation. However, out of the 605 *Nr2f2*-positive NR5A1-deficient SCs, 174 (29%) SCs that co-expressed the male-specific gene *Eif2s3y* (Stévant et al., 2019) could be assigned the ‘male supP-like’ cell identity, 87 (14%) SCs that co-expressed the female-specific gene *Aldh1a2* (Stévant et al., 2019) could be assigned the ‘female supP-like’ cell identity, and 276 (46%) SCs were assigned an ‘intersex supP-like’ cell identity because they co-expressed both *Eif2s3y* and *Aldh1a2* (Fig. 9G). Taken together, these findings indicated that loss of NR5A1 dedifferentiated SCs into a heterogenous population, consisting of pGr-like cells, as well as male, female and intersex E10-E12 supP-like cells.

### Ablation of *Nr5a1* does not alter ATP metabolism in SCs

Previously, it was shown in adrenocortical cells cultured *in vitro* that suppression of NR5A1 reduces the production of the energy carriers adenosine triphosphate (ATP) and nicotinamide adenine dinucleotide phosphate (NADPH), and decreases the expression of genes involved in glucose metabolism (Baba et al., 2014). Consistent with this possibility, functional analysis of upregulated genes in NR5A1-deficient SCs highlighted ATP metabolism and glycolysis/gluconeogenesis (Fig. S5; Table S3). However, although the expression of most of the genes involved in ATP synthesis was indeed increased by 20-50% in mutant SCs (Fig. S13A), we found that ATP production was decreased by approximately 20%, although the difference was not statistically significant [(43.7±5.3)×10^4^ relative fluorescence units (RFU) in mutants (*n*=6) versus (35.3±8.1)×10^4^ RFU in controls (*n*=6); *P*=0.060] (Fig. S13B). The association between reduced ATP production and increased gene expression may reflect the fact that SCs were dying. Regarding the glycolysis pathway, the expression of most genes was increased by 20-30% in mutant SCs (Fig. S13C), except that of *Hk1*, *Aldoa*, *Pgam1* and *Eno1*, which was decreased by 20-40% (Fig. S13D). In this context, it is worth noting that these four genes contain NR5A1-responsive elements (Baba et al., 2014), suggesting that their reduced expression was directly linked to the loss of NR5A1. We did not test whether these changes caused an increase or a decrease in glycolysis. The fact that the NR5A1-deficient SCs were dying added a confounding factor, which may affect the correct interpretation of the result, as was the case for ATP production.

## DISCUSSION

**Fig. 10.**
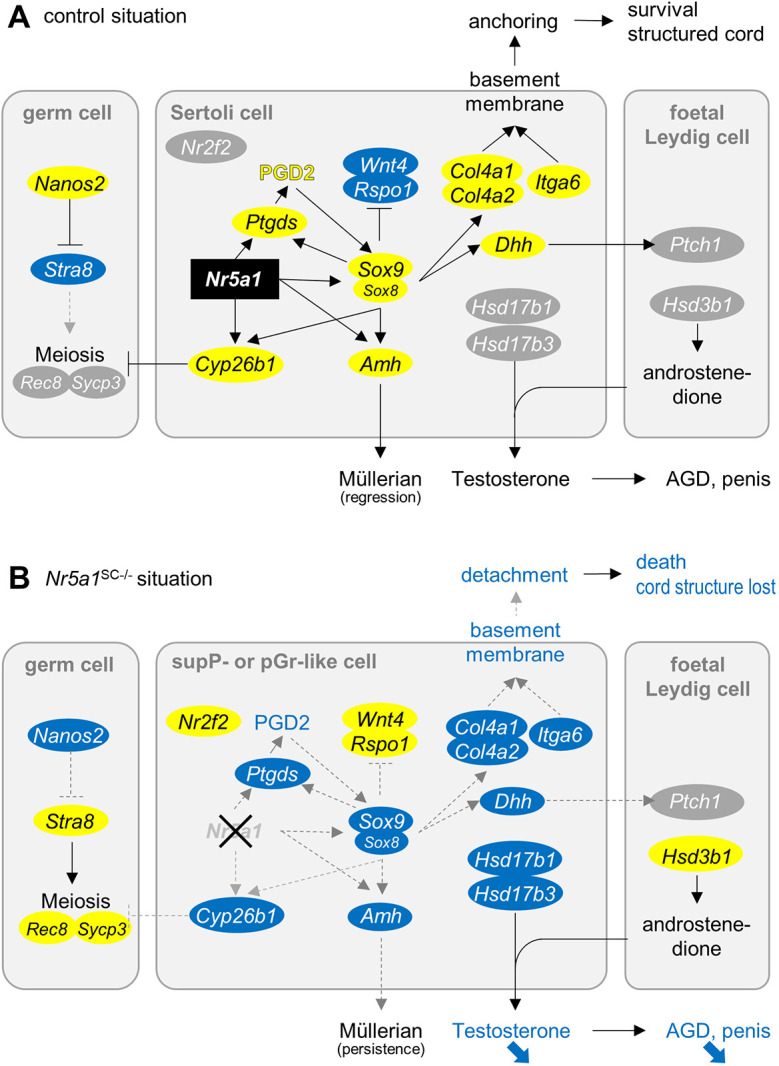
**Summary of the alterations induced by the loss of NR5A1 in SCs after the sex determination period.** (A) Control situation. (B) *Nr5a1*^SC−/−^ situation. Blue and yellow ovals are downregulated and upregulated genes, respectively. The decreased expression of *Sox9*, *Amh*, *Cyp26b1* and *Dhh* in *Nr5a1*^SC−/−^ testes can be ascribed to the loss of NR5A1 in SCs as these genes contain NR5A1-binding sites in their regulatory regions (De Santa Barbara et al., 1998; Arango et al., 1999; Sekido and Lovell-Badge, 2008; Kashimada et al., 2011; Li et al., 2014). AGD, anogenital distance; PGD2, prostaglandin D2; pGr, pre-granulosa; supP, supporting progenitor.

Understanding DSDs provides tools and insights into more general developmental processes. In this study, we investigated the role of the DSD gene *Nr5a1* in SCs after the sex determination period. Our study shows that SCs lacking NR5A1 from E13.5 onwards lose the expression of genes that define or maintain SC identity, illustrating that NR5A1 plays a primary role in these processes. Among the genes that have reduced expression in SCs lacking NR5A1 is *Dmrt1*, a pioneer factor that enables SOX9 binding and plays a crucial role in post-natal male sex maintenance (Matson et al., 2011; Lindeman et al., 2021). However, as *Dmrt1* knockout does not alter SC fate until PND7 (Murphy et al., 2022), it is unlikely that this gene participates in the sex reversal displayed by *Nr5a1*^SC−/−^ males. Most of the defects displayed by *Nr5a1*^SC−/−^ testes are schematized in Fig. 10. The set of abnormalities generated by ablation of *Nr5a1* at distinct timepoints in other studies are recapitulated in Table S5.

### NR5A1-deficient SCs change their cellular identity

In the absence of NR5A1, some SCs gain expression of genes characteristic of the supP-like lineage (e.g. *Nr2f2*), and some undergo sexual transdifferentiation into pGr-like cells. In general, there are two possible scenarios for transdifferentiation: either the cells first dedifferentiate into an intermediate state of limited potency and then redifferentiate into another cell type, or the cells transform directly into another cell type. The first scenario is not known to occur in other mouse genetic models of sexual transdifferentiation (Jiménez et al., 2021). In the case of *Foxl2* post-natal ablation, transdifferentiation of granulosa cells into SCs is immediate (Uhlenhaut et al., 2009), favoring the second scenario. In the present case, we cannot claim that the NR5A1-deficient SCs first reverted to the supP-like state, which would correspond to the intermediate state, before acquiring the pGr-like state. It should be noted, however, that the supP-like cells do not revert to a true bipotential state, as defined previously (Stévant et al., 2019). A large proportion of these cells do not follow a strict male-female binary distribution. These cells express both male- and female-specific genes, and are therefore defined as intersex supP-like cells. Some of them retain a male-oriented identity, whereas others display a female-oriented identity. Such a distribution, coupled with the presence of pGr-like cells, suggests a scenario in which NR5A1-deficient SCs could first dedifferentiate into male-oriented supP-like cells, which then become intersex supP-like cells because they lose the expression of male-promoting genes and express female-promoting genes, then progressively become female-oriented supP-like cells, and finally differentiate into pGr-like cells.

Interestingly, ablation of *Nr5a1* at an earlier stage than in the present study (i.e. from E12.5) allows SCs to acquire a FOXL2-positive granulosa cell identity, resulting in male-to-female sex reversal (Ikeda et al., 2021). This indicates that SCs devoid of NR5A1 from E12.5 can fully transdifferentiate into granulosa cells, whereas those that lose NR5A1 from E13.5 (our study) are no longer licensed to do so. Conversely, when ablation of *Nr5a1* occurs at a later stage than in the present study (i.e. from E14.5), SCs do not change identity at all, but a proportion of them die by MDM2/TRP53-dependent apoptosis (Anamthathmakula et al., 2019). In the present study, ablation of *Nr5a1* at E13.5 causes SCs to revert to supP-like and pGr-like cells that do not reach a state of full sexual transdifferentiation as they remain FOXL2 negative. Thus, the earlier NR5A1 is lost in SCs after sex determination, the greater is the cellular plasticity that SCs retain, suggesting that *Nr5a1* locks SC identity over time.

### SCs lacking NR5A1 from E13.5 die by anoikis

Cells sense their position and maintain their adhesion through specific interactions with the ECM. This plays a major role in the regulation of various processes, including cell survival and maintenance of seminiferous cord integrity (Matoba et al., 2008; Barrionuevo et al., 2009; Georg et al., 2012; Chen et al., 2013). This function is mediated by integrins, which are the cell membrane receptors that interact with components of the ECM. They recruit nonreceptor tyrosine focal adhesion kinases (FAKs), which are activated in response to adhesion (Lu and Rounds, 2012). Disruption or loss of integrin-ECM adhesion impairs cell survival and often leads to detachment-induced cell death, a process also known as anoikis (Gilmore, 2005; Vachon, 2011). Physiologically, anoikis serves to prevent detached cells from adhering to an ECM other than that to which they were assigned, or to prevent cells from migrating to wrong locations. Similar to apoptosis, anoikis leads to DNA fragmentation and activation of caspase 3 (Tedja et al., 2021) but, unlike apoptosis, anoikis does not require the involvement of TRP53 (McGill et al., 1997).

We report here that the expression of *Itga6*, an integrin subunit crucial for fetal testis organization (Fröjdman and Pelliniemi, 1994), and *Ptk2b*, a member of the FAK family (Beverdam et al., 2010) is reduced in NR5A1-deficient SCs. We also show that the adhesion molecules and ECM expressed by NR5A1-deficient SCs are profoundly altered, with some of the acquired proteins being characteristic of pGr cells (e.g. *Col18a1*, *Fn1*, *Nid2* and *Dcn*; Table S2) (Piprek et al., 2018). Thus, it is conceivable that all these changes disrupt the anchorage of SCs to the ECM and induce death by anoikis, thereby altering the integrity of the testis cord epithelium. Consistent with this proposal, we show that SCs lacking NR5A1 display the hallmarks of anoikis: they express many anoikis-related genes, they are TUNEL positive (i.e. their DNA is fragmented), they have increased caspase 3 activity and they die even when TRP53 is absent. In addition, using the MSC-1 cell line as a surrogate model for SCs, we show that impairment of cell attachment *in vitro* induces SC death.

Interestingly, when NR5A1 is deleted from E12.5, SCs do not die but transdifferentiate into granulosa cells (Ikeda et al., 2021). When NR5A1 is lost from E13.5, SCs change their identity to supP-like and pGr-like cells and die by anoikis (our study). When SCs lose NR5A1 later than E14.5, many of them survive, giving rise to testes with well-defined seminiferous tubules at adulthood, but others die by a TRP53-dependent mechanism (Anamthathmakula et al., 2019). This observation suggests that the ability of SCs to die and the underlying mechanisms depend on both NR5A1 and the cell differentiation status.

### Death of NR5A1-deficient SCs affects genital tract development

The action of AMH on regression Müllerian ducts in males proceeds rostro-caudally and begins as early as E13.5 (Staack et al., 2003; Mullen and Behringer, 2014). As *Amh* expression is lost only from E14.5 in *Nr5a1*^SC−/−^ mutants, AMH production starts normally and then gradually decreases to zero after E14.5. It is therefore not surprising that the rostral part of the Müllerian ducts regresses normally, making their derivatives (oviducts and anterior portions of the uterine horns) absent in the mutants, whereas the caudal part of the Mullerian ducts persists beyond E14.5, being at the origin of the posterior portions of the uterine horns, the body of the uterus and the vagina of adult *Nr5a1*^SC−/−^ mutants. A similar outcome is described in mice lacking MDM2 in SCs from E14.5 (Fouchécourt et al., 2016).

In addition, *Nr5a1*^SC−/−^ mutants display normal Wolffian duct-derived genitalia (epididymis, vas deferens and seminal vesicles) (Zhao and Yao, 2019). These develop under the influence of testosterone, the synthesis of which starts as early as E13.5 in mice (Livera et al., 2006). Their presence indicates therefore that *Nr5a1*^SC−/−^ mutants are exposed to testosterone during fetal development. This is surprising as SCs are progressively lost and, with them, is also lost the ability to convert fetal LC-produced androstenedione into testosterone through the expression of *Hsd17b1* and *Hsd17b3* (Shima et al., 2013). Thus, another enzyme necessarily compensates for the loss of *Hsd17b1* and *Hsd17b3*, as considered elsewhere (Rebourcet et al., 2020). In this regard, HSD17B12 is a good candidate as it has been shown to convert androstenedione to testosterone (Blanchard and Luu-The, 2007). If so, a cell type distinct from fetal LCs should be involved because LCs are not able to produce testosterone from androstenedione (Shima et al., 2013). This production is nevertheless insufficient as AGD and penile bone length, both of which are highly sensitive to testosterone levels between E14.5 and E17.5 in mice (Welsh et al., 2014), are reduced in *Nr5a1*^SC−/−^ mutants.

### Differentiation of the other testicular cell types when SCs lack NR5A1

Regarding the fate of GCs in *Nr5a1*^SC−/−^ testes, the data show that most of them become meiotic and die. This may seem strange given that the expression of the meiotic suppressor genes *Fgf9* and *Fgfr1* (Bowles et al., 2010) is increased in NR5A1-deficient SCs (Table S2). However, the fact that *Cyp26b1* expression is completely lost in NR5A1-deficient SCs may explain, on its own, why GCs enter meiosis. In fact, CYP26B1 is necessary in SCs to prevent meiotic initiation and to maintain GCs in an undifferentiated state (Li et al., 2009). In addition, the expression of the quiescence-inducing gene *Nanos2* (Saba et al., 2014) is reduced in GCs (Table S2). Thus, not only is meiotic initiation not properly prevented, but mitotic quiescence of GCs may also be inappropriately promoted in *Nr5a1*^SC−/−^ testes.

As for fetal LCs, their specification and/or development is dependent on SCs, in particular due to DHH- and PDGF-dependent signaling pathways (Yao et al., 2002; Brennan et al., 2003). Our finding that fetal LCs are present in *Nr5a1*^SC−/−^ testes, despite reduced expression of *Dhh* and *Pdgfa* in SCs, suggests that these pathways are no longer required beyond E13.5 to allow proper emergence or survival of fetal LCs. Our findings are consistent with the persistence of LCs in mouse models with SCs genetically ablated from E14.5 by using the diphtheria toxin approach (Rebourcet et al., 2014; Wang et al., 2020). Furthermore, the fetal LCs of *Nr5a1*^SC−/−^ testes appear to be functional as testicular transabdominal descent occurs, suggesting normal INSL3 production (Nef and Parada, 1999; Verma-Kurvari et al., 2005).

PTM cells require that SCs produce DHH and ECM for their proper differentiation (Clark et al., 2000; Pierucci-Alves et al., 2001). Accordingly, their survival is compromised when SCs are ablated at E14.5 (Rebourcet et al., 2014; Wang et al., 2020). Thus, the loss of SCs, the changes in the ECM they produce and the decrease in *Dhh* expression in *Nr5a1*^SC−/−^ testes called into question the fate of the PTM cells. As a matter of fact, the expression of hallmark genes such as *Acta2*, *Tagln* and *Tpm1* (Jeanes et al., 2005; Sohni et al., 2019) is reduced in PTM cells of *Nr5a1*^SC−/−^ mutants (Table S2). Correspondingly, ACTA2 expression appeared to be reduced or even lost in PTM cells, notably in those surrounding the seminiferous cords where AMH expression is decreased or lost in the SCs (Fig. S14). This suggests that PTM cells are affected in *Nr5a1*^SC−/−^ testes.

Knowing that PTM cells and adult LCs derive from a single population of *Wnt5a*^+^ steroidogenic progenitors (Ademi et al., 2022) and that *Wnt5a* expression is reduced in *Nr5a1*^SC−/−^ mutants (Table S2), it is conceivable that the alteration of PTM cells during fetal development and the absence of adult LCs in PND60 testes are linked. It is also worth noting that very few adult LCs, if any, develop in *Pdgfa*^−/−^ mutants (Gnessi et al., 2000). The reduction of *Pdgfa* expression in SCs lacking NR5A1 (Table S2) may therefore be related to the absence of adult LCs in *Nr5a1*^SC−/−^ mutants. Further investigation is required to address these issues.

## MATERIALS AND METHODS

### Mice

Mice were housed in a licensed animal facility (agreement #C6721837). They were on a mixed C57BL/6 (50%)/129/SvPass (50%) genetic background. All experiments were approved by the local ethical committee (Comité d'Ethique en Expérimentation Animale de l'IGBMC, Com'Eth, accreditation APAFIS #18323- 2018113015272439_v3), and were supervised by N.B.G., M.M. and N.V., who are qualified in compliance with the European Community guidelines for laboratory animal care and use (2010/63/UE). The *Nr5a1* conditional allele (*Nr5a1*^tm1.1Ics^, also called *Nr5a1*^L2/L2^) was established at the Institut Clinique de la Souris (*i*CS, Illkirch, France), in the context of the French National Infrastructure for Mouse Phenogenomics PHENOMIN (http://www.phenomin.fr). Noon of the day on which a vaginal plug was detected was taken as E0.5. All fetuses were collected by caesarean section. Adult mice (PND60) were anesthetized by intraperitoneal injection of a lethal anesthetic mixture made of xylasin (3 mg/ml) and ketamine (20 mg/ml), and tissues were immediately fixed by intracardiac perfusion of 4% (w/v) paraformaldehyde (PFA) dissolved in PBS. With the exception of scRNA-seq experiments (see below), *Plekha5*^Tg(AMH-cre)1Flor^;*Nr5a1*^+/+^;*Gt(ROSA)26Sor*^tm1(EYFP)Cos^ and *Plekha5*^Tg(AMH-cre)1Flor^;*Nr5a1*^L2/L2^; *Gt(ROSA)26Sor*^tm1(EYFP)Cos^ males are referred to as controls and *Nr5a1*^SC−/−^ mutants, respectively. Generation and genotyping of mice is described in the supplementary Materials and Methods.

### Blood sample collection and testosterone measurement

After anesthesia with a lethal dose of xylasin and ketamine as described above, the blood of PND60 adult mice was collected into heparinized Microvette tubes (Sarstedt, Nümbrecht, Germany) by intra-cardiac sampling. The tubes were then centrifuged at 5000 ***g*** for 5 min and the resulting plasma samples were frozen until further use. Testosterone concentrations were determined by enzyme-linked immunosorbent assay using a commercially available kit (AR E-8000, LDN, Labor Diagnostika Nord, Nordhorn, Germany). Statistical significance was further assessed using bilateral, unpaired Student's *t*-tests.

### Morphology, histology, IHC and *in situ* hybridization

Following collection, E12.5-E15.5 fetuses and tissues were fixed for 16 h in 4% (w/v) PFA at 4°C or in Bouin's fluid at 20°C. After removal of the fixative, samples were rinsed in PBS and placed in 70% (v/v) ethanol for long-term storage, external morphology evaluation and organ weight measurement. They were then embedded in paraffin and 5 µm-thick sections were made using a Leica Biosystems RM2245 microtome. For histology, sections were stained with Hematoxylin and Eosin (H&E).

For IHC, antigens were retrieved for 1 h at 95°C either in 10 mM sodium citrate buffer at pH 6.0 or in Tris-EDTA at pH 9.0 [10 mM Tris base, 1 mM EDTA and 0.05% (v/v) Tween 20]. Sections were rinsed in PBS, then incubated with appropriate dilutions of the primary antibodies (Table S6) in PBS containing 0.1% (v/v) Tween 20 (PBST) for 16 h at 4°C in a humidified chamber. After rinsing in PBST (three times for 3 min each), detection of the bound primary antibodies was achieved for 45 min at 20°C in a humidified chamber using Cy3-conjugated or Alexa Fluor 488-conjugated antibodies (Table S7). Nuclei were counterstained with 4′,6-diamidino-2-phenyl-indole (DAPI) diluted at 10 µg/ml in the mounting medium (Vectashield; Vector Laboratories, Newark, CA, USA). ImmPRESS Polymer Detection Kits (MP-7500, Vector Laboratories) were used according to the manufacturer's protocol. Confocal microscopy, light-sheet microscopy and image processing used for vasculature analysis are described in the supplementary Materials and Methods. The surface area occupied by YFP-positive cells was measured using a macro command designed for Fiji software [Imaging Center of Institut de Génétique et de Biologie Moléculaire et Cellulaire (IGBMC)]. Data were expressed as a percentage of YFP-positive surface area relative to the surface area of the entire testis section. At least four samples were analyzed per genotype. Statistical analysis was done by a bilateral, unpaired Student's *t*-test, assuming equal variances after arcsine transformation of the percentages.

For detection of *in situ* hybridization, a commercially available kit (RNAscope 2.5 HD Reagent kit–RED, 322360, Advanced Cell Diagnostics) was used according to the manufacturer's instructions. Briefly, deparaffinized sections from PFA-fixed E13.5 fetuses were treated with hydrogen peroxide for 10 min, washed, and boiled at 100°C in 1× Target Retrieval Reagent for 10 min. Next, Protease IV was applied for 15 min at 40°C on dehydrated sections and slides were then washed in distilled water. The pre-warmed probes (RNAscope Probe-Mm-Wnt4, 401101, and RNAscope Probe-Mm-Fst, 454331, Advanced Cell Diagnostics) were applied on the sections for 2 h at 40°C. The slides were washed in 1× wash buffer and were subjected to a series of signal amplification (AMP1 to AMP8) according to the manufacturer's instructions (RNAscope 2.5 HD Reagent kit-RED). Hybridization signals were detected using a chromogenic Fast RED-B/Fast RED-A reagent. The presence of *Wnt4* and *Fst* mRNA was identified as red punctate dots. The sections were counterstained for 3 min with 12.5% (v/v) Harris Hematoxylin diluted in distilled water.

### BrdU incorporation and TUNEL assays

Bromodeoxyuridine (BrdU; Sigma-Aldrich, Saint-Quentin-Fallavier, France) dissolved at 5 mg/ml in PBS was injected intraperitoneally in pregnant females at 50 mg/kg of body weight. Two hours later, fetuses were collected (E13.5-E15.5), fixed and embedded as described above. BrdU incorporation was detected in 5 µm-thick sections using an anti-BrdU mouse monoclonal antibody (diluted 1:100, Table S6) and indirect IHC as described above. At least three samples per stage and per genotype were analyzed. Data were expressed as percentages of the numbers of BrdU-positive cells relative to the numbers of DDX4-positive cells. Statistical analysis was done by a bilateral, unpaired Student's *t*-test, assuming equal variances after arcsine transformation of the percentages.

TUNEL-positive cells were detected on sections from PFA-fixed samples using the *In Situ* Cell Death Detection Kit, Fluorescein (Roche, Mannheim, Germany), according to the manufacturer's instructions. At least three samples were analyzed per genotype. Data were expressed as the ratio between the numbers of TUNEL-positive cells quantified on entire sections and the surface areas of the testis sections (μm^2^). Statistical significance was assessed by using bilateral, unpaired Student's *t*-tests.

### Real-time RT-qPCR analyses of RNA extracted from whole testes

Fetal testes were dissected, isolated from mesonephros, snap frozen in liquid nitrogen and stored at −80°C until use. Whole-testis total RNA was extracted using RNeasy Mini Kit (Qiagen, Les Ulis, France). RT-qPCR was performed on 5 ng RNA aliquots using Luna Universal One-Step RT-qPCR Kit (New England Biolabs, Evry, France), according to the manufacturer's instructions. The primers used are listed in Table S8. Triplicates of at least four samples were used for each genotype at each stage. The relative transcript levels were determined using the ΔΔCt method, and normalized to *Acta*, the expression of which is not affected by ablation of *Nr5a1*.

### Purification of YFP-positive SCs

To dissociate cells, the testes of controls and mutants were incubated for 10 min at 37°C in 350 μl of trypsin/EDTA (0.05%, w/v), phenol red solution (Gibco Invitrogen, Auckland, New Zealand), filtered through a 70 μm cell strainer to generate single-cell suspensions, centrifuged at 3000 ***g*** and suspended in 300 µl PBS as described (Stévant et al., 2018). The YFP-positive and YFP-negative cells were sorted separately by FACS using an Aria II flow cytometer (BD Biosciences, Le Pont de Claix, France). Sorted cell suspensions were then lysed for overnight at 55°C in a proteinase K-containing buffer [100 mM Tris-HCl (pH 8), 100 mM EDTA, 250 mM NaCl, 1% SDS, containing 1 mg/ml proteinase K], and DNA was extracted and genotyped by PCR using standard protocols and primers as indicated above.

### Cell preparation for scRNA-seq

Testes from E13.5 and E14.5 *Nr5a1*^L2/L2^ (control) and *Plekha5*^Tg(AMH-cre)1Flor^;*Nr5a1*^L2/L2^; *Gt(ROSA)26Sort*^m1(EYFP)Cos^ (mutant) fetuses were dissected out in PBS and cell suspensions were prepared as described above. Cell number and viability were determined by a Trypan Blue exclusion assay on a Neubauer Chamber. Samples consisting of >90% viable cells were processed on the Chromium Controller from 10× Genomics (Leiden, the Netherlands). Ten thousand cells were loaded per well to yield approximately 5000 to 6000 captured cells into nanoliter-scale Gel Beads-in-Emulsion (GEMs, 10x Genomics). Details about library preparation, sequencing and data processing are described in the supplementary Materials and Methods.

### Analysis of anoikis and measurement of ATP concentration

A total of 914 anoikis-related genes was acquired from GeneCards (https://www.genecards.org), and 88 of these protein-coding genes were selected based on a score >2.0. The overlap of DEGs in *Nr5a1*-deficient SCs and anoikis-related genes was visualized using the R package ‘VennDiagram’ (https://cran.r-project.org/src/contrib/VennDiagram_1.7.3.tar.gz). The caspase activity was determined at E13.5 on eight (control) and six (NR5A1-deficient) batches of about 5000 FACS-purified SCs each, using the Caspase-Glo 3/7 assay (Promega, USA). The ability of MSC-1 SCs to die from anoikis when their attachment was impaired was tested using an anoikis assay kit (ab284938, Abcam, UK). Briefly, cells were seeded at a density of 30,000 cells/well on normal (uncoated) and anchorage-resistant (hydrogel-coated) plates and incubated for 24 h. Live cells were detected with the 3-(4,5-dimethylthiazol-2-yl)-2,5-diphenyltetrazolium bromide (MTT, a tetrazolium dye) assay or by using calcein acetoxymethyl (calcein AM) fluorescence, whereas dead and dying cells were detected using the ethidium homodimer (ethD-1) fluorescent dye, according to the manufacturer's instructions (ab284938, Abcam).

Measurement of ATP levels was performed using six batches of approximately 5000 FACS-purified SCs each, isolated from control and *Nr5a1*^SC−/−^ E13.5 testes. The cells were lysed in a lysis buffer [2 mM 1,4-dithiothreitol; 25 mM Tris-phosphate (pH 7.8); 2 mM 1,2-diaminocyclohexane-N,N,N′,N′-tetraacetic acid; 1.25 mg/ml lysozyme; 2.5 mg/ml bovine serum albumin; 10% (v/v) glycerol; 1% (v/v) Triton X-100] and ATP was quantified using the ATP determination kit (A22066, Invitrogen, USA) according to the manufacturer's instructions.

## Supplementary Material

10.1242/develop.201710_sup1 Supplementary information

Table S1. Cluster identities

Table S2. List of deregulated genes in *Nr5a1*^SC-/-^ testes, categorized by cell-types

Table S3. List of functional GO terms identified from deregulated genes in *Nr5a1*^SC-/-^ testes, categorized by cell-types

Table S4. List of anoikis-related genes (ARGs) deregulated in the *Nr5a1*^SC-/-^ testis
