# Supplementary material for: Loss of NR5A1 in mouse Sertoli cells after sex determination changes cellular identity and induces cell death by anoikis
Source: Development. 2023 Dec 11;150(24):dev201710. doi: 10.1242/dev.201710 (PMC10753587; doi:10.1242/dev.201710)
Supplement: Supplementary information [file develop-150-201710-s1.pdf]

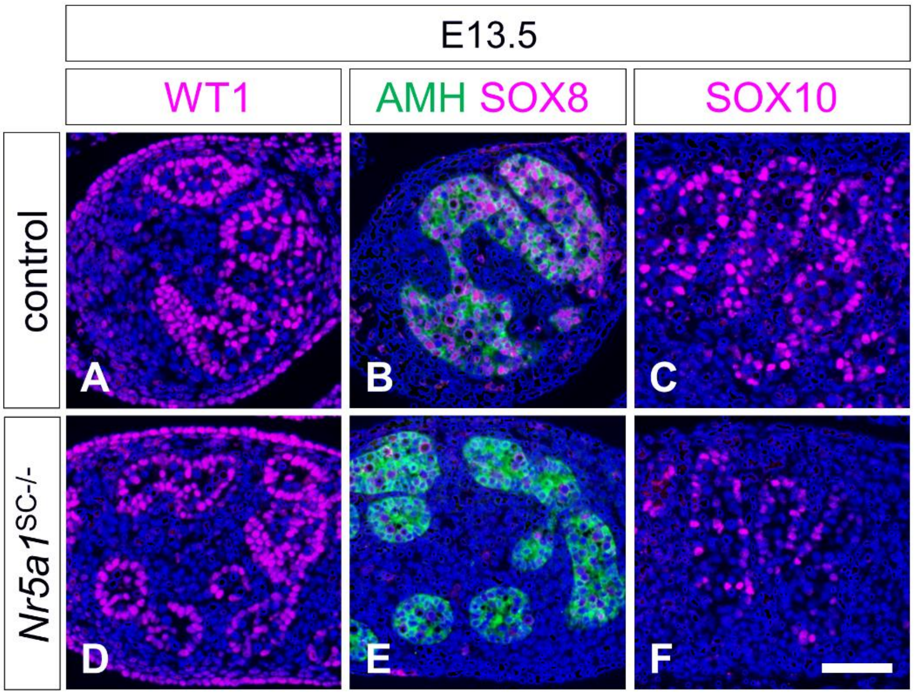

Fig. S1. Ablation of *Nr5a1* in SC impairs SOX8 and SOX10 expression. (A-F) Detection of WT1, SOX8, SOX10 (magenta signals) and AMH (green signals) on histological sections of control (A-C) and *Nr5a1*$^{SC–/–}$ (D-F) testes at E13.5. Nuclei were counterstained with DAPI (blue signal). Scale bar (in F): 50 µm (A-F).

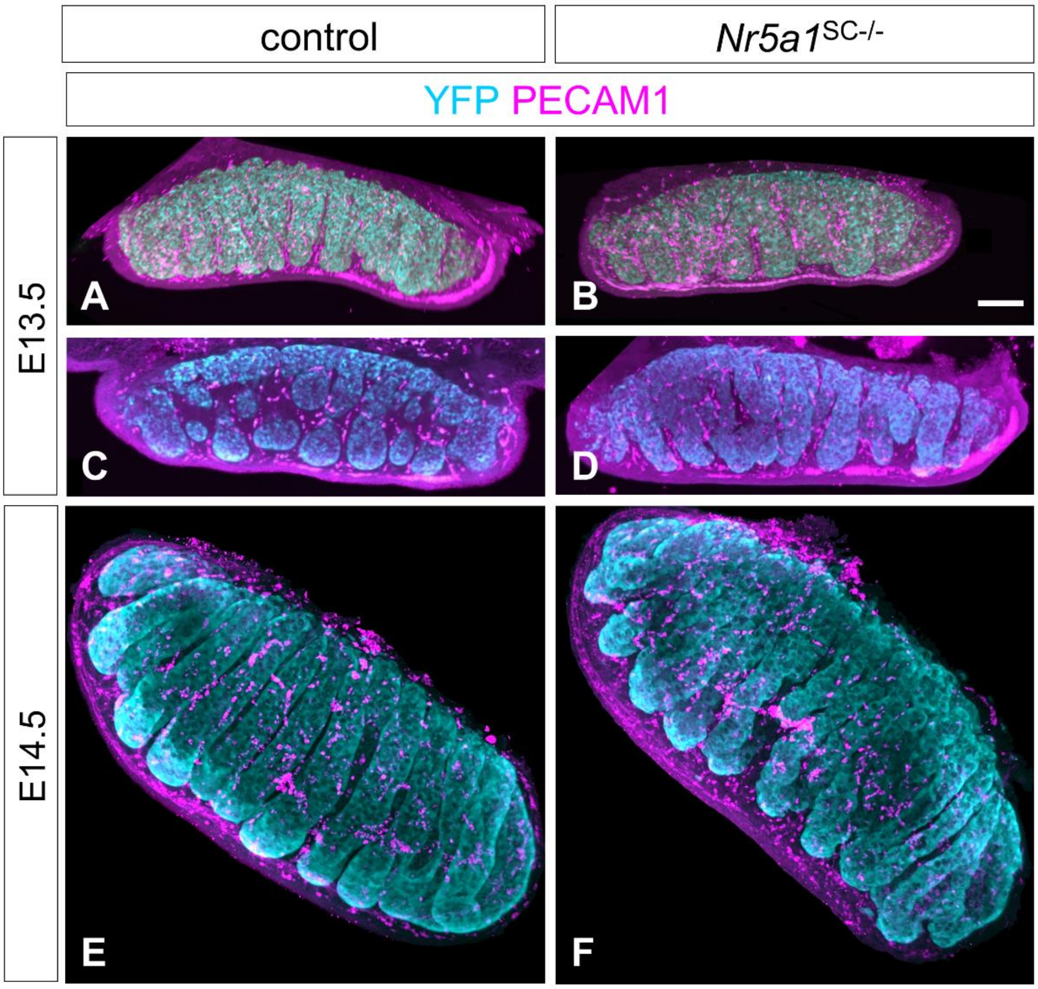

Fig. S2. Ablation of *Nr5a1* in SC does not alter testicular vascularization. (A,B) Detection of YFP in SC (cyan signal) and PCAM1 in vascular cells (magenta signal) using light-sheet microscopy on whole mounts of E13.5 control (A) and *Nr5a1*$^{SC–/–}$ (B) testes. (C,D) Virtual transverse sections made through the testes illustrated in A,B. (E,F) Detection of YFP (cyan signal) and PCAM1 (magenta signal) using confocal spinning disk microscopy on whole mounts of E14.5 control (E) and *Nr5a1*$^{SC–/–}$ (F) testes. The formation of the coelomic artery and of the invading vessels between the seminiferous cords appeared similar between control and *Nr5a1*$^{SC–/–}$testes. Scale bar (in B): 100 µm (A,B), 120 µm (C,D) and 80 µm (E,F).

Development • Supplementary information

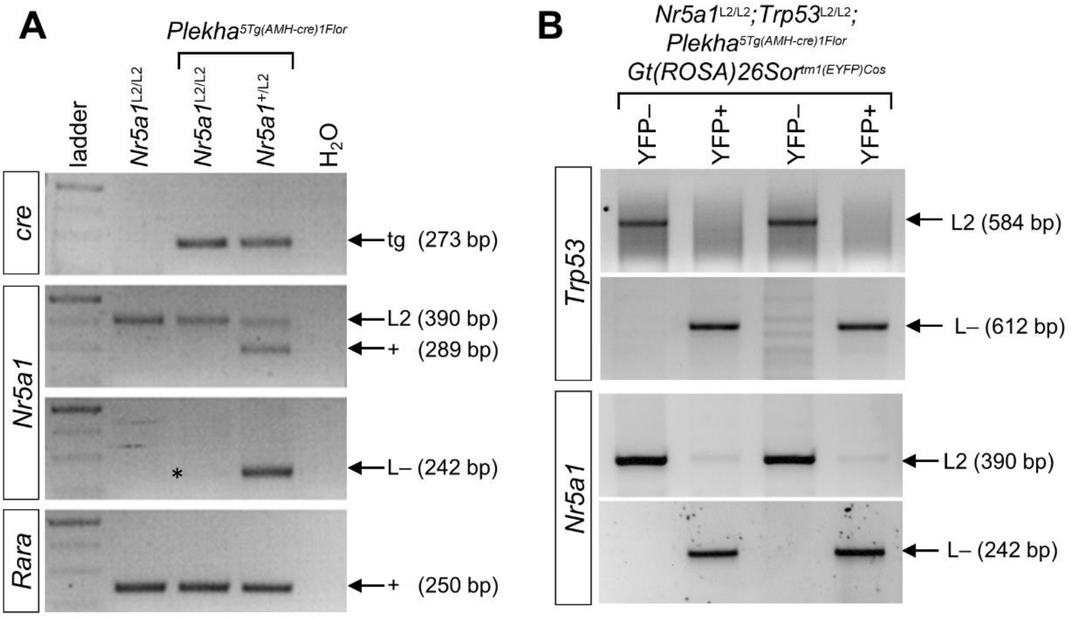

Fig. S3. NR5A1-deficient SC die even in the absence of TRP53. (A) PCR analysis of genomic DNA extracted from control (*Nr5a1*$^{L2/L2}$), mutant (*Plekha5*$^{Tg(AMH\text{-}cre)1Flor}$; *Nr5a1*$^{L2/L2}$) and heterozygote (*Plekha5*$^{Tg(AMH\text{-}cre)1Flor}$; *Nr5a1*$^{+/L2}$) testes of newborns. Upper panel shows genotyping of *Cre* transgene; middle panel shows genotyping of *Nr5a1* alleles; lower panel shows genotyping of *Rara* locus, attesting for an equivalent loading of DNA in each lane. The sizes of the expected fragments are indicated on the right: tg, Cre transgene; L2 and +, *lox*P-flanked and wild-type alleles, L–, excised, null allele. Note that the mutant testis contained only traces of the *Nr5a1* L– allele (asterisk), indicating that SC bearing the excised allele were no longer present at birth. (B) PCR analysis of genomic DNA extracted from FACS-purified YFP-positive (YFP+) and negative (YFP–) cells contained in *Nr5a1*$^{SC-/-}$;*Trp53*$^{SC-/-}$ mutants fetuses at E14.5. Upper panel shows genotyping of *Trp53* alleles; lower panel shows genotyping of *Nr5a1* alleles. The YFP-negative cells (i.e., all cells except SC) contained only the unexcised (L2) alleles, while the YFP-positive cells (i.e., SC) contained only the cre-recombined (L–), null alleles. Note however that traces of the *Nr5a1* L2 alleles were detected in YFP-positive cells.

Development • Supplementary information

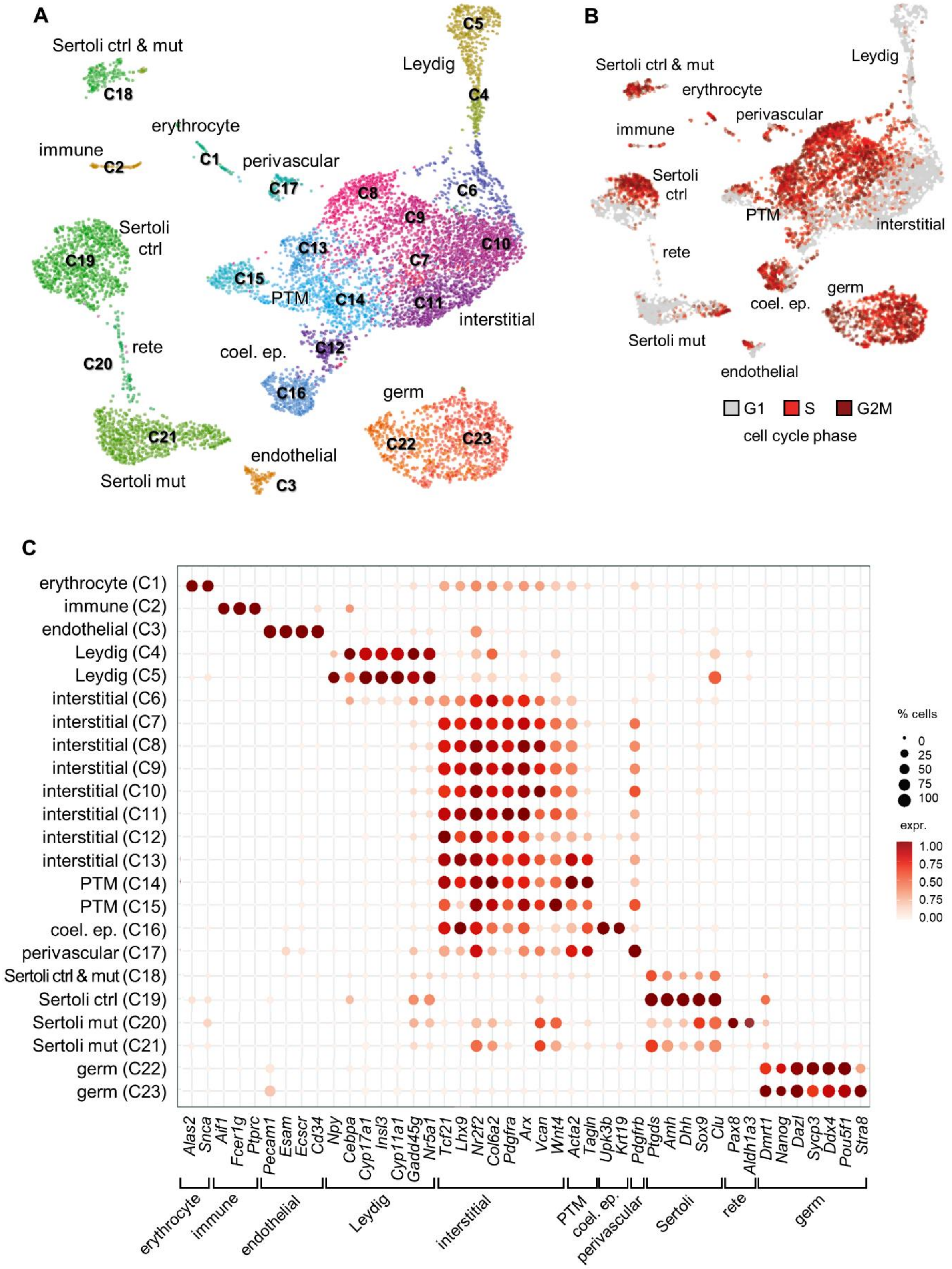

Fig. S4. Identification of cell clusters generated from the single-cell transcriptomes. (A-B) UMAP projection of the 8,998 cells coloured by cell clusters (panel A) or by phases of the cell-cycle, as indicated (panel B). Associated cell annotation is indicated close to the corresponding cell clusters (named C1-23). (C) Dot plot with the expression of selected markers (x axis) for each cell cluster (y axis). The dot size represents the percentage of cells expressing a given gene within a given cell cluster. The colour intensity (from light to dark red) indicates the average expression (log normalized counts) of a given gene within a given cell cluster. Legend: coel. ep., coelomic epithelium; ctrl, control; mut, mutant; PTM, peritubular myoid.

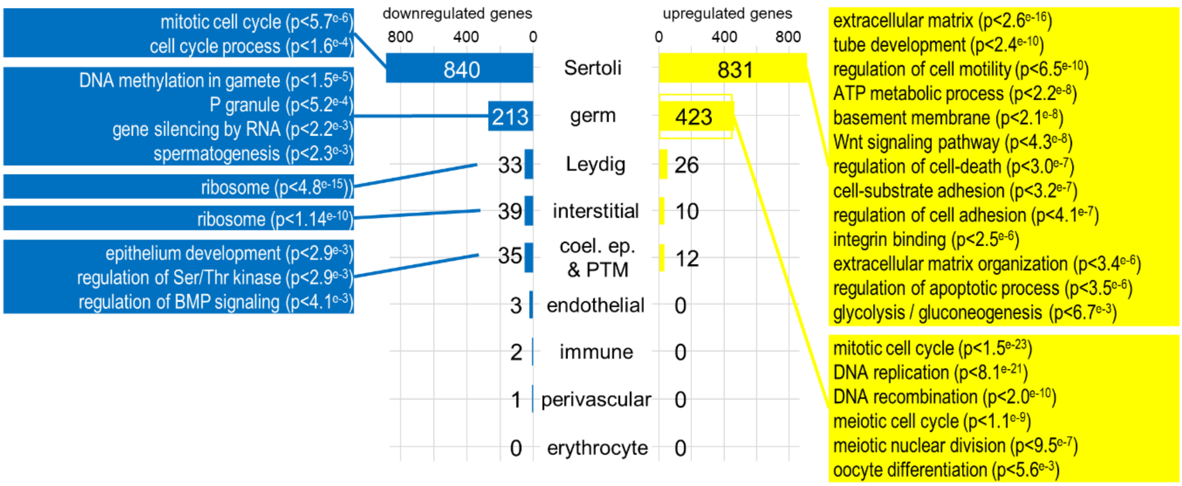

Fig. S5. Number of differentially expressed genes and associated GO terms. Bar plot representing the number of significantly downregulated (left side, in blue) and upregulated (right side, in yellow) genes between control and *Nr5a1*$^{SC-/-}$ cells (x axis), in each cell-type (y axis). Enriched GO terms and their associated *p* values are given for each cell-type. Legend: coel. ep., coelomic epithelium; PTM, peritubular myoid.

Development • Supplementary information

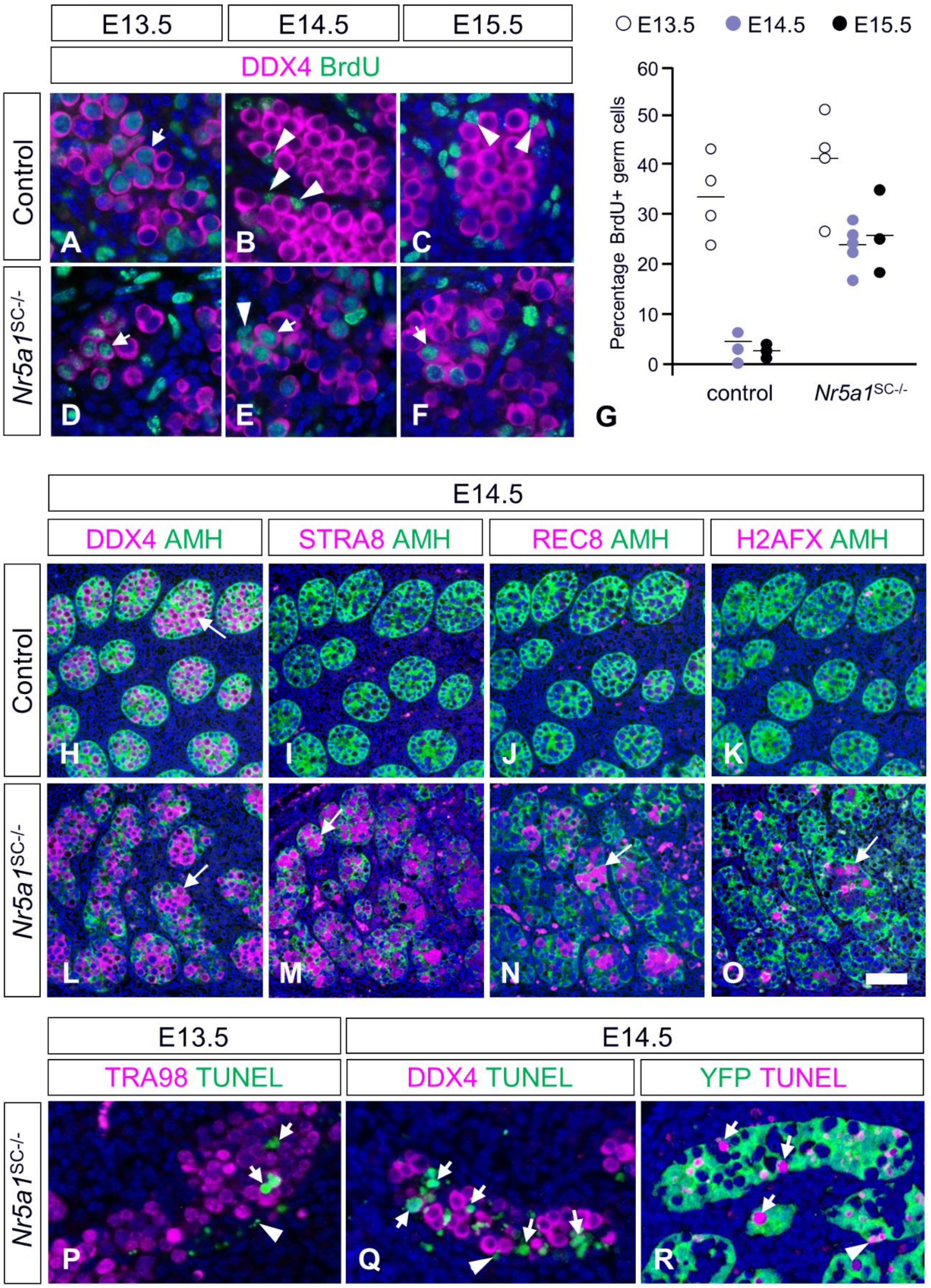

Fig. S6. Germ cells initiate meiosis and die in *Nr5a1*$^{SC–/–}$ mutants. (A-F) Detection of DDX4 (magenta cytoplasmic signal) and BrdU (green nuclear signal) on histological sections of control (A-C) and *Nr5a1*$^{SC–/–}$ (D-F) testes at E13.5 (A,D), E14.5 (B,E) and E15.5 (C,F). Arrows and arrowheads point to BrdU-positive GC and SC, respectively. (G) Dot plots showing the percentage of BrdU-positive GC in control (n=3 to 4) and *Nr5a1*$^{SC–/–}$ (n=3 to 4) testes, as a function of the developmental stages. (H-O) Detection of DDX4, STRA8,

Development • Supplementary information

REC8, H2AFX (magenta signals) and AMH (green cytoplasmic signal) on histological sections of control (H-K) and *Nr5a1*$^{SC-/-}$ (L-O) testes at E14.5. Arrows point to GC. The meiotic proteins STRA8, REC8, H2AFX were detected in the mutant but not in the control testes. Arrows point to GC. (P,Q) Detection of TUNEL-positive cells (green signal) and TRA98- or DDX4-positive GC (magenta signals) on sections from E13.5 (P) and E14.5 (Q) *Nr5a1*$^{SC-/-}$testes. (R) Detection of TUNEL-positive cells (magenta signal) and YFP-positive SC (green signal) on sections from an E14.5 *Nr5a1*$^{SC-/-}$ testis. Arrows point to TUNEL-positive GC, while arrowheads point to TUNEL-positive SC. The total number of TUNEL-positive cells was significantly larger in *Nr5a1*$^{SC-/-}$ than in control testes [79 ± 9 cell/mm^2 (n=6) versus 27 ± 11 cell/mm^2 (n=3), respectively; $p<0.05$]. Nuclei were counterstained with DAPI (blue signal). Scale bar (in O): 10 µm (A-F,P,R), 50 µm (H-O).

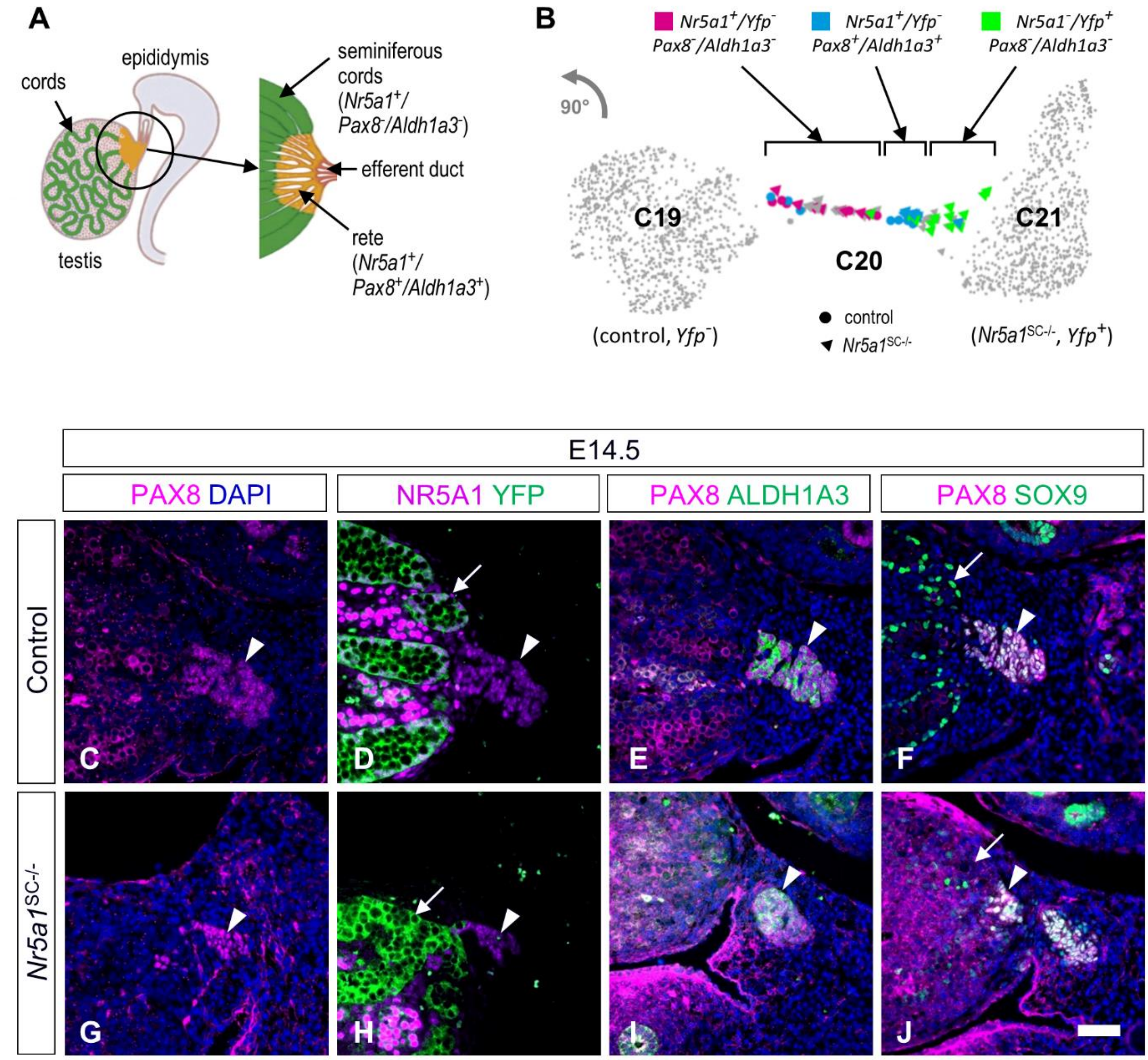

Fig. S7. Cluster C20 corresponds to *rete* testis cells, in which gene excision by cre recombinase is not operational. (A) Diagram illustrating the location and organization of *rete* testis in the mouse. Seminiferous cords are in green, while *rete* testis is in brown. (B) Magnification of UMAP plot (rotated by 90° with respect to Fig. 6) for clusters C19, C20 and C21. Cells belonging to C19 (from control testes, YFP-negative cells) and to C21 (from *Nr5a1*$^{SC–/–}$ testes, YFP-positive) are in grey. Cells belonging to C20 and expressing distinct combination between *Nr5a1*, *Yfp*, *Pax8* and *Aldh1a3* are depicted by a colour code: pink represents cells expressing *Nr5a1* but not *Yfp*, *Pax8* or *Aldh1a3* (SC); blue stands for cells expressing *Nr5a1*, *Pax8* and/or *Aldh1a3*, but not *Yfp* (*rete* testis cells); green represents cells expressing *Yfp* but not *Nr5a1*, *Pax8* or *Aldh1a3* (mutant SC). Circles and triangles represent control and *Nr5a1*$^{SC–/–}$ cells, respectively. Note that the YFP-reporter transgene was absent in the control gonads but present in the *Nr5a1*-deficient gonads used for the scRNA-seq experiments. (C-J) Detection of PAX8 and NR5A1 (magenta signals) together with YFP, ALDH1A3 or SOX9 (green signals) on sections from control (C-F) and *Nr5a1*$^{SC–/–}$ (G-J) testes at E14.5. Note that the YFP-reporter transgene was present in both the control and the

Development • Supplementary information

Nr5a1-deficient gonads used for IHC analysis. Arrows and arrowheads point to SC and rete testis cells, respectively. The rete testis cells (arrowheads) were PAX8- and ALDH1A3-positive (E,I). They were also NR5A1-positive, YFP-negative and SOX9-positive in both control (D,F) and *Nr5a1*$^{SC–/–}$ testes (H,J). In contrast, SC (arrows) were NR5A1-negative, YFP-positive and SXO9-negative in the *Nr5a1*$^{SC–/–}$ testes (H,J), but NR5A1-positive, YFP-negative and SOX9-positive in the control testes (D,F). Nuclei were counterstained with DAPI (blue signal). Scale bar (in J): 50 µm (C-J).

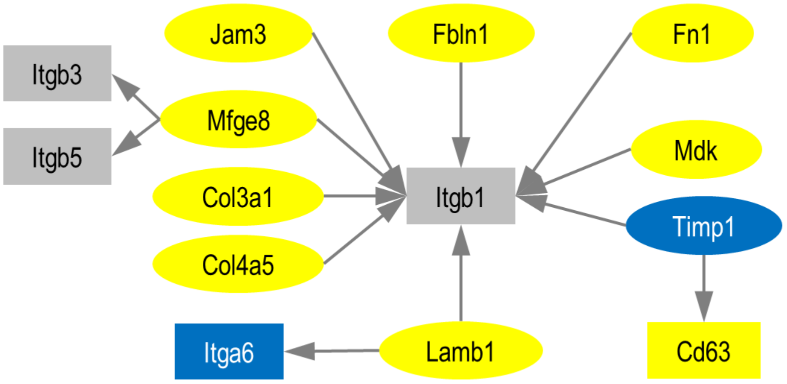

Fig. S8. Selected ligand-receptor interactions between SC and GC. Diagram of the ligand-receptor interactions between SC and GC, as revealed from the single cell transcriptomes. Each node of the network corresponds to a given gene encoding a ligand (oval shape) or a receptor (rectangle shape). Intercellular communication network among SC and GC were predicted using the CellTalkDB database, which contains literature-supported ligand-receptor pairs. Downregulated and upregulated genes are blue and yellow coloured, respectively. Grey boxes indicated receptors whose expression in not changed. The arrows point from the ligand to the receptors.

Development • Supplementary information

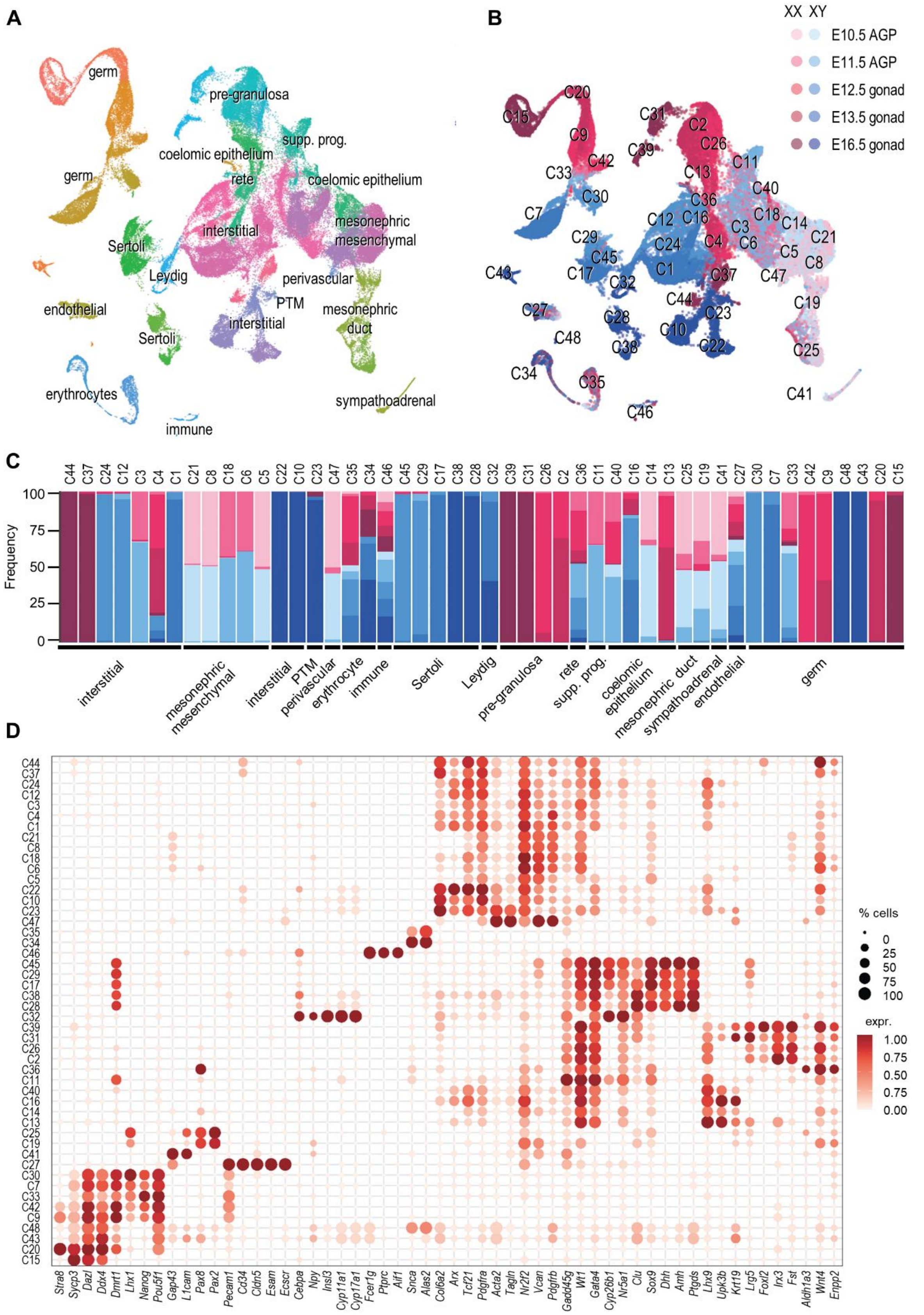

Fig. S9. Identification of cell clusters generated from the atlas. (A-B) UMAP projection of the 94,705 cells from the single-cell transcriptomic atlas of gonad development recently published (Mayère et al., 2022) coloured by cell clusters (in panel A) or

Development • Supplementary information

by developmental stage, from E10.5 to E16.5 as indicated (in panel B). Cell clusters (C1-C48) and their associated cell annotation are indicated. (C) Stacked bar plot showing the proportion of the developmental stages (from light at E10.5 to dark colour at E16.5) amongst the different cell clusters with male (XY, blue) and female (XX, pink) cells. (D) Dot plot with the expression of selected markers (x axis) for each cell cluster (y axis). The dot size represents the percentage of cells expressing a given marker within a given cell cluster. The colour intensity (from light to dark red) indicates the average expression (expr., log normalized counts) of a given marker within a given cell cluster. Legend: AGP, adrenal-gonadal primordium; E, embryonic day; PTM, peritubular myoid cells; supp. prog., supporting progenitor cells; XX, female, XY, male.

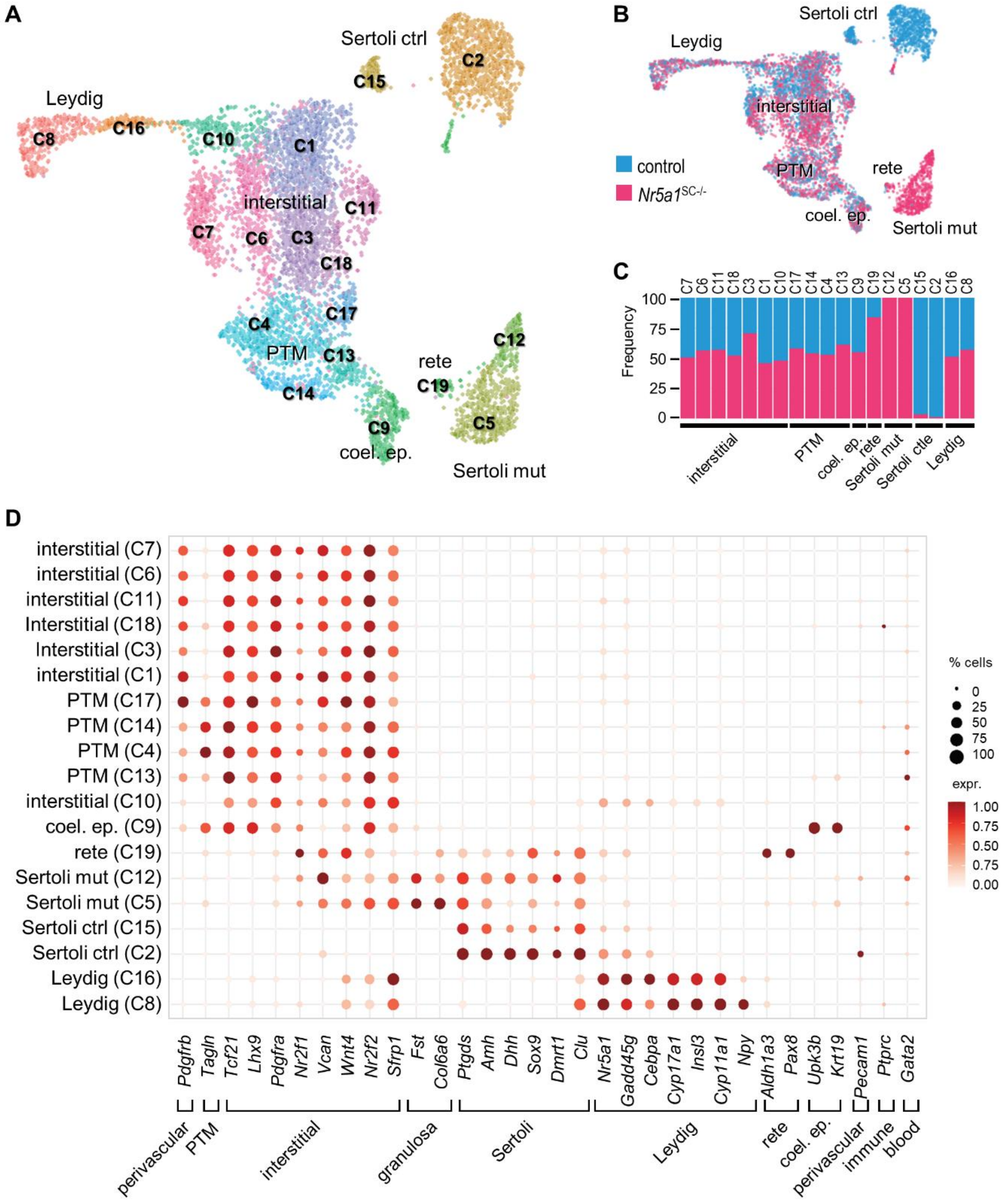

Fig. S10. Identification of cell clusters generated from the single-cell transcriptomes of gonad somatic cells only. (A-B) UMAP projection of the 8,998 cells coloured by cell clusters (panel A) or by genotype, as indicated (panel B). Associated cell annotation is indicated close to the corresponding cell clusters (named C1-C19). (C) Proportions of control and mutant cells in each cluster is indicated as coloured bars. Legend: Legend: coel. ep., coelomic epithelium; ctrl, control; mut, mutant; PTM, peritubular myoid cells. (D) Dot plot with the expression of selected markers (x axis) for each cell cluster (y axis). The dot size represents the percentage of cells expressing a given gene within a given cell cluster. The colour intensity (from light to dark red) indicates the average expression (log normalized counts) of a given gene within a given cell cluster.

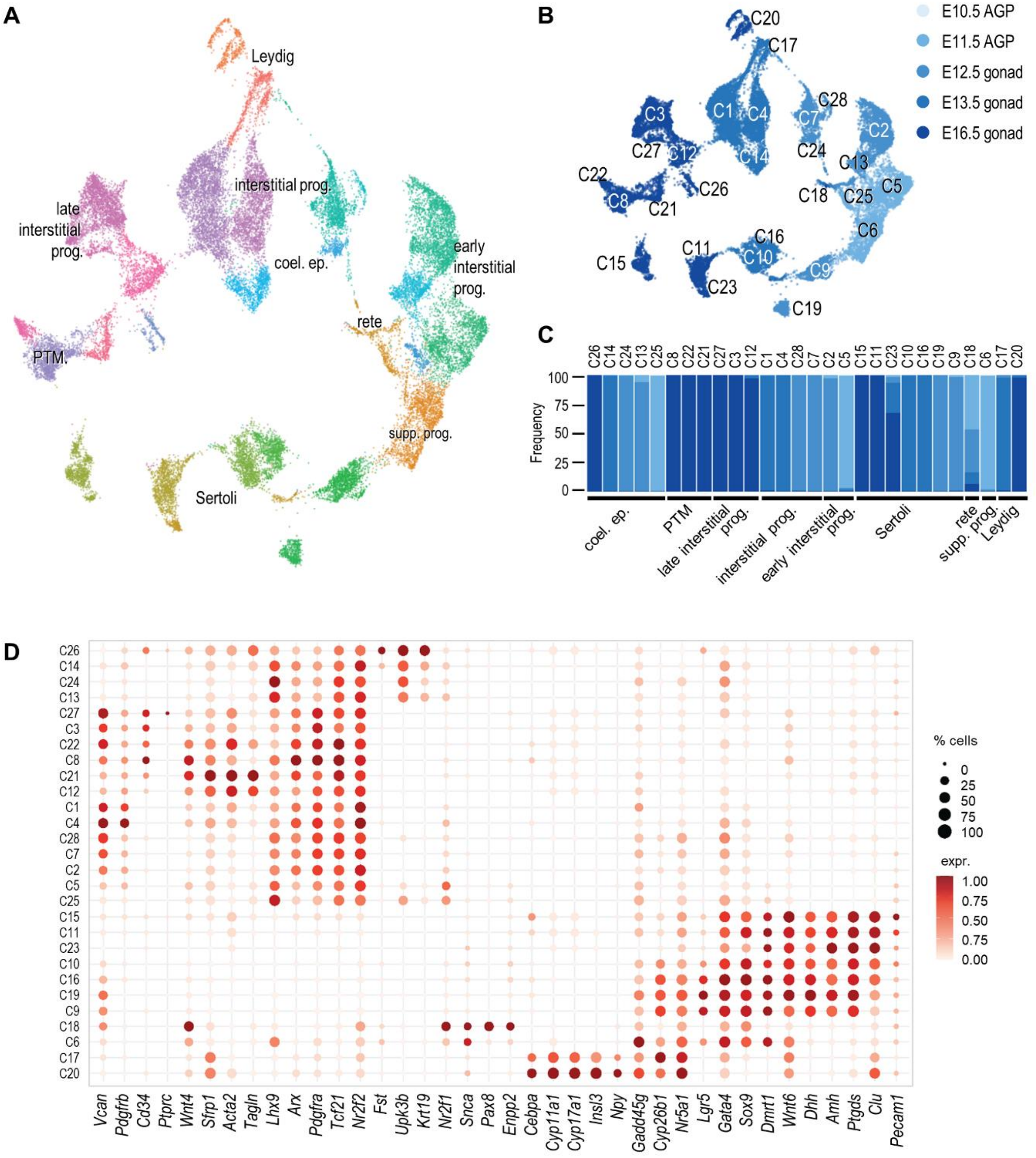

Fig. S11. Identification of somatic male cell clusters generated from the atlas. (A-B) UMAP projection of the male somatic cells from the single-cell transcriptomic atlas of gonad development (Mayère et al., 2022) coloured by cell clusters (in panel A) or by developmental stage, from E10.5 to E16.5 as indicated (in panel B). Cell clusters (C1-C28) and their associated cell annotation are indicated. (C) Stacked bar plot showing the proportion of the developmental stages (from light at E10.5 to dark blue at E16.5) amongst the different male cell clusters. (D) Dot plot with the expression of selected markers (x axis) for each cell cluster (y axis). The dot size represents the percentage of cells expressing a given marker within a given cell cluster. The colour intensity (from light to dark red) indicates the average expression (log normalized counts) of a given marker within a given cell cluster. Legend: AGP, adrenal-gonadal primordium; coel. ep., coelomic epithelium; E, embryonic day; prog., progenitor; PTM, peritubular myoid cells; supp. prog., supporting progenitor cells.

Development • Supplementary information

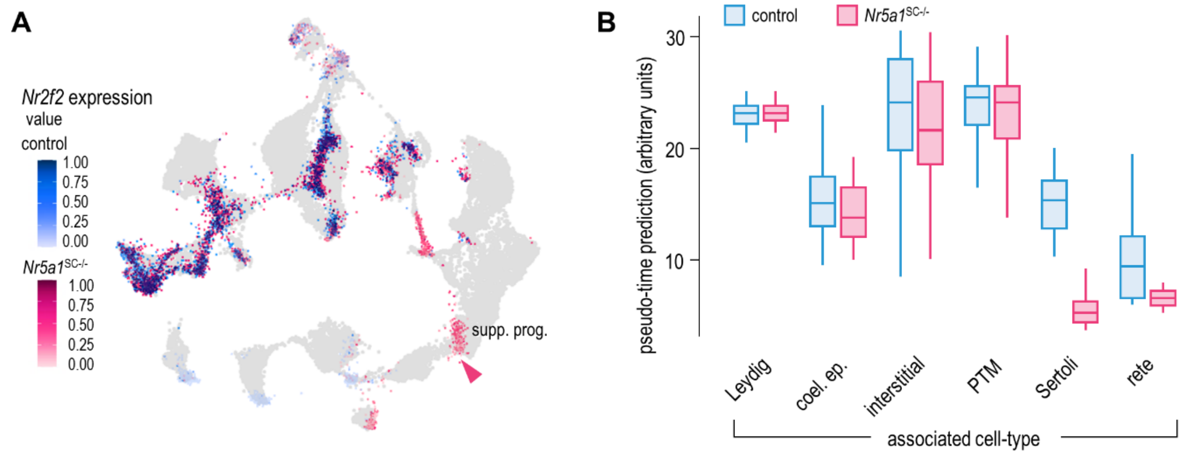

Fig. S12. NR5A1-deficient SC express *Nr2f2* and display a transcriptomic signature corresponding to an earlier stage of development for SC. (A) Expression of *Nr2f2* in control (blue) and *Nr5a1*$^{SC-/-}$ (pink) somatic cells projected on the reference single-cell transcriptomic atlas of male somatic cell development (Mayère et al., 2022). The color intensity (from white to dark blue or dark pink) indicates the level of expression. (B) Boxplot showing the pseudo time prediction for each of the major somatic cell-types of the control (blue boxes) and *Nr5a1*$^{SC-/-}$ (pink boxes) testes. The NR5A1-deficient SC population appears younger than the control SC population. Legend: coel. ep., coelomic epithelium; PTM, peritubular myoid cells; supp. prog., supporting progenitor cells.

Development • Supplementary information

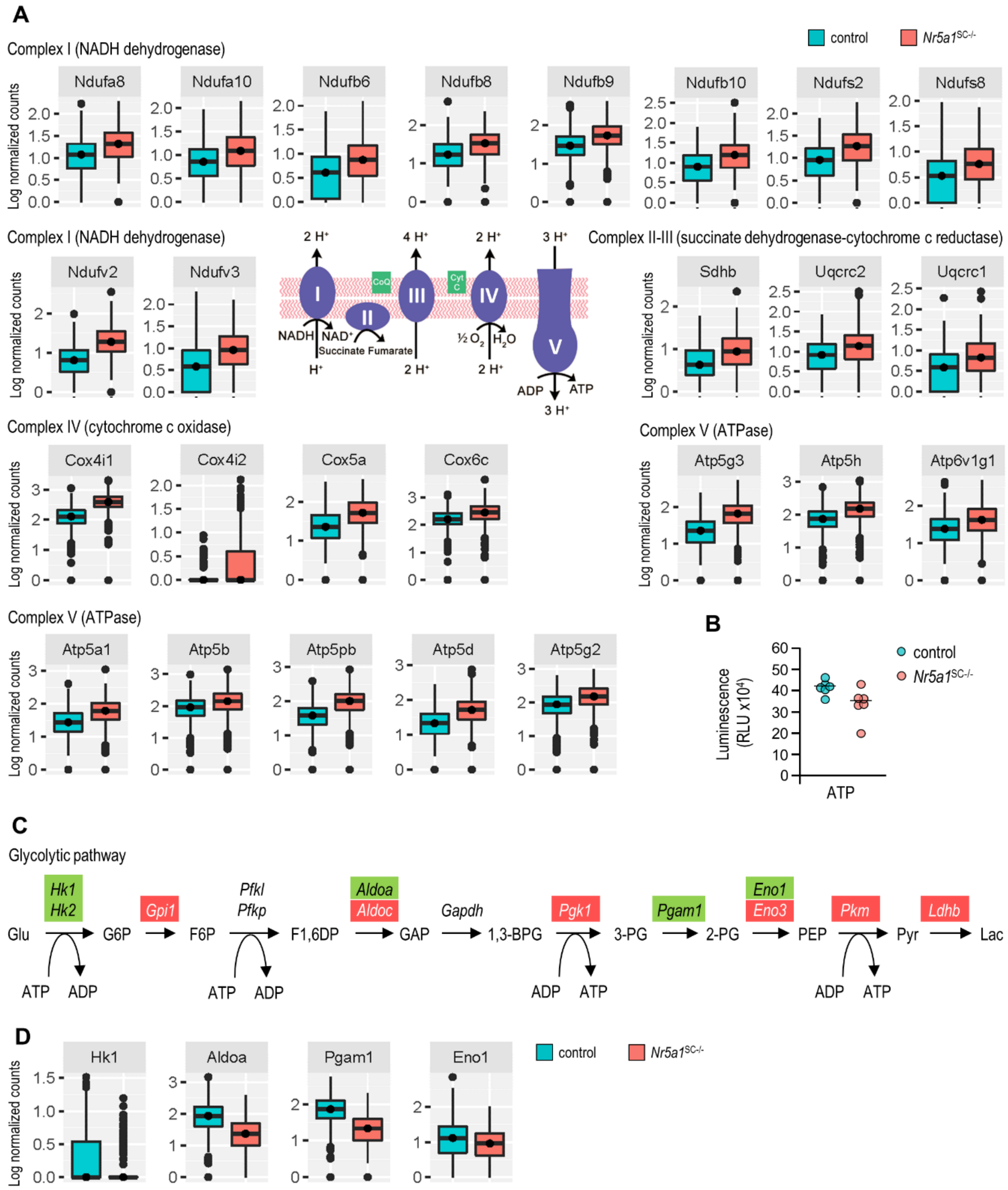

Fig. S13. ATP synthesis is not dramatically altered in NR5A1-deficient SC. (A) Tukey box plots illustrating medians, ranges and variabilities of log normalized expression of the indicated genes belonging to NADH dehydrogenase (complex I of respiratory chain), succinate dehydrogenase (complex II), cytochrome c reductase (complex III), cytochrome c oxidase (complex IV) and ATPase (complex V) in SC of control (blue boxes) and *Nr5a1*$^{SC-/-}$ (pink boxes) testes. A scheme of the respiratory chain is also depicted. (B) ATP quantities measured in of FACS-purified control (blue dots, n =6 batches) and NR5A1-deficient (pink dots, n =6 batches) SC. RLU, relative luminescence units. The difference is not statistically significant. (C) Intermediate metabolites and genes involved in the glycolytic pathway. Genes that were up-regulated and down-regulated in SC from *Nr5a1*$^{SC-/-}$ testes are indicated by red and green boxes, respectively. (D) Tukey box plots illustrating medians,

Development • Supplementary information

ranges and variabilities of log normalized expression of the genes harbouring a NR5A1-reposnive element (Baba et al., 2014) in SC of control (blue boxes) and *Nr5a1*$^{SC–/–}$ (pink boxes) testes. Legend:.1,3-BPG, 1,3-bisphospho-glycerate; 2-PG, 2-phosphoglycerate; 3-PG, 3-phospho-glycerate; ADP Adenosine-5'-diphosphate; ATP, Adenosine-5'-triphosphate; *Aldoa*, aldolase A; *Aldoc*, aldolase C; CoQ, coenzyme Q (ubiquinon); CytC, cytochrome C; *Eno1* and *Eno3*, enolase 1 and 2; F6P, fructose 6-phosphate; F1,6DP, fructose 1,6-bisphosphate; G6P, glucose 6-phosphate; GAP, glyceraldehyde 3-phosphate; *Gapdh*, Glyceraldehyde 3-phosphate dehydrogenase; Glu, glucose; *Gpi1*, phosphoglucose isomerase 1; *Hk1* and *Hk2*, hexokinase 1 and 2; *Ldhb*, lactate dehydrogenase B;.PEP, phospho-enolpyruvate; *Pfkl*, liver phosphofructokinase; *Pfkp*, platelet phosphofructokinase; *Pgam1*, phosphoglycerate mutase 1; *Pgk1*, phosphoglycerate kinase 1; *Pkm*, muscle pyruvate kinase.

Development • Supplementary information

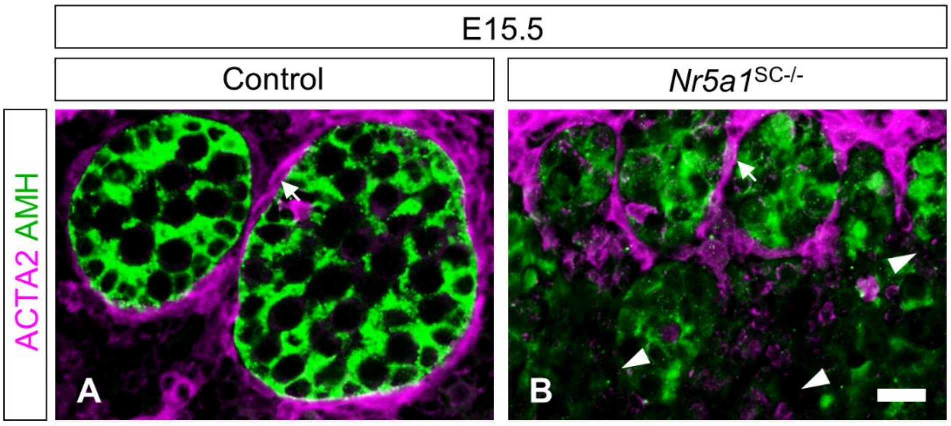

Fig. S14. Ablation of *Nr5a1* in SC impairs ACTA2 expression in peritubular myoid cells. (A-F) Detection of ACTA2 (magenta signal) and AMH (green signal) on histological sections of control (A) and *Nr5a1*$^{SC–/–}$ (B) testes at E15.5. Arrows point to ACTA2-positive peritubular myoid (PTM) cells, whereas arrowheads point to ACTA2-negative PTM cells. Note that ACTA2 expression is lost in PTM cells adjacent to SC where AMH expression is reduced or lost in *Nr5a1*$^{SC–/–}$ testes. Conversely, ACTA2 expression is retained in PTM cells adjacent to AMH-positive SC, which were preferentially located on the mesonephric side of the in *Nr5a1*$^{SC–/–}$ testes. Scale bar (in B): 10 µm.

Table S1. Cluster identities

Available for download at

https://journals.biologists.com/dev/article-lookup/doi/10.1242/dev.201710#supplementary-data

Table S2. List of deregulated genes in *Nr5a1*$^{SC–/–}$ testes, categorized by cell-types

Available for download at

https://journals.biologists.com/dev/article-lookup/doi/10.1242/dev.201710#supplementary-data

Table S3. List of functional GO terms identified from deregulated genes in *Nr5a1*$^{SC–/–}$ testes, categorized by cell-types

Available for download at
https://journals.biologists.com/dev/article-lookup/doi/10.1242/dev.201710#supplementary-data

Table S4. List of anoikis-related genes (ARGs) deregulated in the *Nr5a1*$^{SC–/–}$ testis

Available for download at
https://journals.biologists.com/dev/article-lookup/doi/10.1242/dev.201710#supplementary-data

Table S5. Comparison of the outcome of *Nr5a1* or SC ablation during fetal development according to four distinct studies.

reference	Ikeda et al., 2021	Souali-Crepo et al., present study	Wang et al., 2020	Anamthathmakula et al., 2019
Cre line	Sox9$^{tm3(cre)Crm}$	*Plekha5*$^{Tg(AMH-cre)1Flor}$	*Plekha5*$^{Tg(AMH-cre)1Flor}$	*Tg(Amh-cre)8815Reb*
*lox*P-flanked allele	*Nr5a1*^{tm2Klp}	*Nr5a1*$^{tm1.1Ics}$	*Gt(ROSA)26Sor*$^{tm1(DTA)Jpmb}$	*Nr5a1*^{tm2Klp}
stage of Cre-mediated excision	E12.5	E13.5	E14.5	E15.5
stage of SC-death	ND	E14.5-E15.5.	E14.5	E14.5 to E15.5 (partial)
mechanism of SC-death	–	anoikis	diphtheria toxin	MDM2/TRP53-dependent apoptosis
AMH expression	lost E13.5	lost E13.5-E14.5	lost E14.5	E15.5 to E16.5
SOX9 expression	lost E13.5	lost E14.5	ND	lost E17.5-E18.5
FOXL2 expression	acquired E13.5	No	ND	ND
seminiferous cord disruption	E12.5	E14.5	E14.5	E16.5-E17.5
ECM defects	laminin lost E14.5	col-IV lost E14.5	laminin lost E14.5-E16.5	ND
fate of GC	meiotic E15.5	meiotic E14.5<br>dead E15.5-E16.5	dead E16-5-E18.5	dead E15.5-E18.5
fate of fetal LC	reduced number E13.5	not affected	proliferating E16.5-E18.5	not affected
fate of adult LC	–	absent	ND	ND
fate of PTM cells	ND	ACTA2 lost E15.5	ACTA2 lost E16.5-E18.5	ND
gonad at adulthood	ovary or ovotestis	testis<br>devoid of SC and GC	ND	testis<br>devoid of GC
Müllerian derivatives	complete tract	partial tract	ND	No

ND, not determined

Development • Supplementary information

Table S6. Primary antibodies used for immunohistochemistry experiments.

**Antibodies**	**Species**	**Dilution**	**Source**	**Reference**	**Batch #**
ACTA2	Mouse monoclonal	1/100	Sigma	A5228	056M4828V
ALDH1A1	Rabbit polyclonal	1/50	Abcam	ab52492	GR41450-32
ALDH1A3	Rabbit polyclonal	1/20	Sigma	HPA046271	C106318
AMH	Goat polyclonal	1/100	Santa-Cruz	sc-6886	F0515
BHMT	Rabbit polyclonal	1/200	Anticorps-enligne.fr	ABIN310161	ARP41475-T100
BrdU	Rat monoclonal	1/100	Bio Rad	OBT0030S	B05D250
COL-IV	Rabbit polyclonal	1/50	Abcam	ab19808	GR3399907-2
DDX4	Rabbit polyclonal	1/2000	Abcam	ab13840	GR3317403-1
FOXL2	Goat polyclonal	1/250	Abcam	ab5096	GR181817-20
GATA 4	Goat polyclonal	1/50	Santa-Cruz	sc-1237	D3013
H2AFX	Mouse monoclonal	1/500	Millipore	05-636 (JBW301)	2506483
HSD3B1	Rabbit polyclonal	1/1000	Trans Genic's Inc	KO607	TG030317
NR2F2	Mouse monoclonal	1/200	R&D Systems	PP-H7147-00	A-2
NR5A1	Rabbit polyclonal	1/100	Cosmo Bio	KAL-KO611	TG200616
PAX8	Mouse monoclonal	1/10	Abcam	ab53490	GR3281722-12
PCP4	Rabbit polyclonal	1/500	Sigma Aldrich	HPA005792	03561
PECAM1	Goat polyclonal	1/200	R&D Systems	AF3628	YZU0122121
REC8	Rabbit monoclonal	1/200	Abcam	ab192241	GR3254930-2
SOX8	Mouse monoclonal	1/100	Novus Biologicals	H00030812-M01	07187-8G7
SOX9	Rabbit polyclonal	1/1000	Sigma Aldrich	ab5535	3677685
SOX10	Goat polyclonal	1/1000	R&D Systems	AF2864	VRY081902B
STRA8	Rabbit polyclonal	1/1000	Abcam	ab49602	GR3975217-1
TMEM184A	Rabbit polyclonal	1/200	Best et al., 2008	Best et al., 2008	not applicable
TRA98	Rat monoclonal	1/500	Abcam	ab82527	GR32800356-1
TRP53	Rabbit polyclonal	1/200	Santa-Cruz	sc-6243	A3113
TUBB3	Mouse monoclonal	1/1000	Covance	MMS-435P-0100 (TUJ1 clone)	B354040
WT1	Mouse monoclonal	1/500	Cell Marque	348M-94	31417
YFP	Chicken polyclonal	1/500	Aves	GFP-1020	GFP879484

Development • Supplementary information

Table S7. Secondary antibodies used for immunohistochemistry experiments.

Antibodies	Species	Dilution	Source	Reference	Batch #
Goat anti-rabbit Cy3	Goat	1/500	Jackson Immuno Research	111-165-144	108069
Donkey anti-goat Cy3	Donkey	1/500	Jackson Immuno Research	705-165-147	134527
Goat anti-rat Cy3	Goat	1/500	Jackson Immuno Research	112-165-167	29758
Donkey anti-mouse Cy3	Donkey	1/500	Jackson Immuno Research	715-165-151	167342
Goat anti-rabbit Alexa Fluor 488	Goat	1/500	Interchim	A-11034	1073084
Donkey anti-goat Alexa 488	Donkey	1/500	Interchim	A-11055	2301114
Donkey anti-rat Alexa Fluor 488	Donkey	1/500	Jackson Immuno Research	712-546-153	145823
Donkey anti-chicken Alexa Fluor 488	Donkey	1/500	Jackson Immuno Research	703-545-155	164925

Table S8. Primers used for quantification of mRNA levels by real-time RT-qPCR.

Gene	Accession number	Forward primer sequence	Reverse primer sequence	Amplicon size (bp)
Amh	NM_007445.3	5’-TCCTACATCTGGCTGAAGTGATATG-3’	5’-CAGGTGGAGGCTCTTGGAACT-3’	66
Col6a6	NM_001102607.1	5’-TGATTGCGTTCAGCAACGTG-3’	5’-TCCGGCTTGCATAGATCATAG-3’	157
Cyp26b1	NM_001177713.1	5’-CGACATCCACCGCAACAAG-3’	5’-GGCCTCCTGATATACATTGATGG-3’	151
Dhh	NM_007857.5	5’-GCACAGGATTCACTCCACTACGA-3’	5’-CCAGTGAGTTATCAGCTTTGACC-3’	172
Fst	NM_001301373.1	5’-CTGCTGCTACTCTGCCAGTT-3’	5’-ACATCCTCCTCGGTCCATGA-3’	167
Hsd17b3	NM_008291.3	5’-ATGGAGTCAAGGAGGAAAGGC-3’	5’-ATGGAGTCAAGGAGGAAAGGC-3’	76
Inhbb	NM_008381.4	5’-GCGTCTCCGAGATCATCAGC-3’	5’-CACCTTGACCCGTACCTTCC-3’	188
Itga6	NM_001277970.1	5’-GCGGCTACTTTCACTAAGGACT-3’	5’-TTCTTTTGTTCTACACGGACGA-3’	92
Pcp4	NM_008791.3	5’-GCGACCAACGGAAAAGACAA-3’	5’-TTCAGGTGGACCAGGAAGCA-3’	194
Ptgds	NM_008963.3	5’-GCTCTTCGCATGCTGTGGAT-3’	5’-GCCCCAGGAACTTGTCTTGTT-3’	118
Sox8	NM_011447.3	5’-ACCCGCATCTCCATAACGCA-3’	5’-TGGTGGCCCAGTTCAGTACC-3’	214
Sox9	NM_011448.4	5’-CAGCAAGACTCTGGGCAAG-3’	5’-TCCACGAAGGGTCTCTTCTC-3’	63
Tubb3	NM_023279.3	5’-CGTGAAGTCAGCATGAGGGA-3’	5’-TCCAAGTCCACCAGAATGGC-3’	218

Supplementary Materials and Methods

Mice

To construct the targeting vector a 1.9 kb-long DNA fragment encompassing exon 7 (ENSMUSE00000 693512) was amplified by PCR using 129/SvPass genomic DNA and cloned into an *i*CS proprietary vector containing a *lox*P site, as well as a *lox*P- and *FRT*-flanked neomycin resistance cassette (step1 plasmid). Then, 3 kb- and 3.7 kb-long fragments corresponding to 5' and 3' homology arms were amplified by PCR and introduced into step1 plasmid to generate the targeting construct. This linearized construct was electroporated into 129/SvPass mouse embryonic stem (ES) cells. After selection, targeted clones were identified by PCR using external primers and confirmed by Southern blots (5' and 3' digests) hybridized with neomycin, 5' and 3' external probes. One positive ES clone was injected into C57BL/6J blastocysts. To remove the selection cassette from the *Nr5a1* locus, chimeric males were crossed with *Gt(ROSA)26Sor*$^{tm1(FLP1)Dym}$ females (Farley et al., 2000). Germline transmission was obtained, and a further breeding step was needed to segregate animals bearing the *Nr5a1* L2 allele (also called *Nr5a1*$^{tm1.1Ics}$) from animals bearing the transgene. To inactivate *Nr5a1* in SC, female mice bearing *Plekha5*$^{Tg(AMH\text{-}cre)1Flor}$ (Lécureuil et al., 2002) and *Gt(ROSA)26Sor*$^{tm1(EYFP)Cos}$ (Srinivas et al., 2001) transgenes, and heterozygous for the L2 allele of *Nr5a1* were mated with males heterozygous or homozygous for L2 alleles of *Nr5a1*. The resulting *Plekha5*$^{Tg(AMH\text{-}cre)1Flor}$;*Nr5a1*$^{+/+}$; *Gt(ROSA)26Sor*$^{tm1(EYFP)Cos}$ and *Plekha5*$^{Tg(AMH\text{-}cre)1Flor}$;*Nr5a1*$^{L2/L2}$; *Gt(ROSA)26Sor*$^{tm1(EYFP)Cos}$ males are referred to as control and *Nr5a1*$^{SC\text{-}/\text{-}}$ mutant fetuses, respectively. To test for the role of TRP53, the L2 allele of *Trp53* gene (Jonkers et al., 2001) was further introduced in the mice described above.

Yolk sacs or tail biopsies were taken for DNA extraction. Primers 5'-GTCAAGCGCCCCATGAATGC-3' and 5'-TTAGCCCTCCGATGAGGCTG-3' were first used to amplify *Sry* gene (230 bp-long fragment) for male sex determination. Then, primers 5'-TGAGCCCTGGCACATCCCTCC-3' and 5'-CCTCTGCCCTGCAGGCTTCTG-3' were used to detect *Plekha5*$^{Tg(AMH\text{-}cre)1Flor}$ transgene (273 bp-long amplicon), and primers 5'-AAGGGAGCTG CAGTGGAGTA-3' and 5'-GCCAGAGGCCACTTGTGTAG-3' to detect *Gt(ROSA)26Sor*$^{tm1(EYFP)Cos}$ reporter (520 bp-long amplicon). Primers 5'-CTGTCTCCTGTCTTCTACTACCCTG-3' and 5'-AGCCATTTCAACAGTGCCCCTTCC-3' were used to amplify wild-type (+, 290 bp-long) and

Development • Supplementary information

L2 (400 bp-long) alleles of *Nr5a1*, while primers 5'-GTGGCACATGCATTAGTCCACTTGG-3' and 5'-AGCCA TTTCAACAGTGCCCCTTCC-3' were used to amplify the excised, null, L– (243 bp-long) allele. Primers 5'-CACAAAAACAGGTTAAACCCAG-3' and 5'-AGCACATAGG AGGCAGAGAC-3' were used to amplify the wild-type (288 bp-long) and L2 (370 bp-long) alleles of *Trp53*. Primers 5'-CACAAAAACAGGTTAAACCCAG-3' and 5'-GAAGACAGAAAAG GGGAGGG-3' were used to amplify the excised, null, L– (612 bp-long) allele of *Trp53*. The PCR conditions were 30 cycles with denaturation at 95°C for 30 seconds, annealing at 61°C for 30 seconds and elongation at 72°C for 30 seconds. The amplicons were resolved on 1.5% (w/v) agarose gels, stained by ethidium bromide and visualized under UV light, using standard protocols.

Confocal microscopy, light-sheet microscopy and image analysis

For vasculature imaging, E13.5 embryonic testes were fixed for 4 hours in 4% (w/v) paraformaldehyde, washed in PBS containing 1% (v/v) Triton X-100 and 1% (v/v) dimethyl sulfoxide (DMSO). They were incubated at 4°C for 4 days in PBS containing 0.1% (v/v) Triton X-100 (PBS-X), 1% (v/v) DMSO, 5% (v/v) heat-inactivated normal goat serum, 2% (v/v) heat-inactivated fetal calf serum and the primary antibodies (Table S5). The testes were then washed for 1 day in PBS-X, and incubated for 2 days at 4°C with appropriate Cy3-conjugated or Alexa Fluor 488-conjugated secondary antibodies. They were washed in PBS-X and incubated at room temperature 2 times for 3 hours in clearing solution (Miltenyi Biotec), and 1 hour in ethyl cinnamate. For confocal spinning disk microscopy, cleared testes where mounted onto an Attofluor™ Cell Chamber dish (ThermoFisher Scientific) with a #1.5 cover glass bottom. A Leica 20x/NA 0.75 HC PL APO CS2 objective was used for image acquisition. The following excitation laser lines and detection filter sets were respectively used for the GFP (488 nm, 525-550 nm) and the Cy3 (561 nm, 609-654 nm) labels on a Yokogawa CSU-W1 confocal scanner unit mounted on a Leica DMi8 inverted stand. Stack were acquired with a z-step of 1 µm and 0.322 µm lateral sampling on a Hamamatsu ORCA-Flash4.0 V3 camera. For light sheet microscopy, the testes were embedded in 2% (w/v) low melting point agarose before being cleared. Images were acquired on a Zeiss Lightsheet LS7 equipped with two LSFM 10x/NA 0.2 illumination objectives and an EC Plan-NeoFluar 5x/NA 0.16 detection objective. The excitation lasers line and filters sets were the following for GFP: laser line 488 nm, detection filter 505-545 nm and for Cy3 laser line 561 nm, detection filter 575-615 nm. The calculated light sheet thickness at the waist was 5.67 µm. The stacks were acquired with a z-step of 2.02 µm and 0.54 µm lateral sampling on a Zeiss Axiocam 702 camera. Images where processed in Fiji (Schindelin et al., 2012) for the GFP/Cy3 channels registration and Imaris (Oxford Instruments) for creating the 3D reconstruction of the figures.

Single cell RNA sequencing and data processing

Gel Beads-in-Emulsion (GEMs) were generated by combining barcoded gel beads, a reverse transcription master mix containing cells, and partitioning oil onto Chromium Chip B. Following full length cDNA synthesis and barcoding from poly-adenylated mRNA, GEM were broken and pooled before cDNA amplification by PCR using 11 cycles. After enzymatic fragmentation and size selection, sequencing libraries were constructed by adding Illumina P5 and P7 primers (Illumina, Evry, France), as well as sample index via end repair, A tailing, adaptor ligation and PCR with 10 cycles. Library quantifications and quality controls were determined using Bioanalyzer 2100 (Agilent Technologies, Santa Clara, CA, USA). Libraries were then sequenced on Illumina HiSeq 4000^{TM} as 100 bases paired-end reads, using standard protocols.

Development • Supplementary information

Sequencing data were processed using 10X Genomics software (https://support.10xgenomics.com/). Fastq files were processed with CellRanger (v6.0) on a chimeric genome composed of mm10 *Mus musculus* assembly and 753 bp-long YFP sequence (European Nucleotide Archive accession number AGM20711) (Aliye et al., 2015). The resulting count matrices were aggregated with the Read10X function implemented in Seurat (v4.0.1) (Hao et al., 2021). Cells with less than 200 detected genes and genes detected in less than 10 cells were removed. Doublets were filtered out independently in each individual matrix by using the DoubletFinder R package (v.2.0.2) (McGinnis et al., 2019). Subsequently, the Single-Cell Analysis Toolkit for Gene Expression Data in R (scater v1.10.1) was used to remove outlier cells by using several cell features including the proportions of reads mapping mitochondrial and ribosomal genes, the number of genes and UMIs per cell (McCarthy et al., 2017). Data were normalized using the NormalizeData and the SCT function implemented into Seurat by regressing out the unwanted variation due to cell cycle and to the proportion of mitochondrial genes. The top-3000 most varying genes were used to perform a principal component analysis with the RunPCA function implemented in Seurat. Cells were then clustered by using Seurat graph-based clustering (FindNeighbors and FindClusters functions) on the top-30 principal components, with default parameters. Finally, we used the Uniform Manifold Approximation and Projection (UMAP) method (RunUMAP function) to project cells in a 2D space. Cell clusters were annotated using a set of known marker genes. The FindAllMarkers function implemented in Seurat was used to identify significantly differentially expressed genes (DEGs) between cell clusters. Gene ontology (GO) and pathway enrichment analysis was conducted for each gene expression cluster using the AMEN suite of tools (Chalmel and Primig, 2008) with an BH-adjusted p value of ≤ 0.05. The current dataset was mapped on top of a reference atlas of gonadal development (Mayère et al., 2022) using the FindTransferAnchors and MapQuery functions with default settings. To address this issue, the reference atlas was first processed by using the same pipeline.

For the focused analysis of male gonadal somatic cells, we first selected relevant clusters (i.e. clusters associated with gonadal somatic cell populations such as coelomic epithelial cells, interstitial cells, Leydig cells, rete cells, Sertoli cells, SC) identified from the primary analysis of the current and reference datasets and independently re-analysed the two resulting datasets using the same pipeline. To order male gonadal somatic cells from the reference dataset in pseudotime and infer trajectories over developmental stages, we used the getLineages followed by the getCurves functions using default parameters implemented in the slingshot R package (version 1.8.0) (Street et al., 2018) by indicating the C6 cell cluster (the earliest cluster associated with supporting progeniotor cells) as starting cells and C20 (Leydig cells), C3 (late interstitial cells), C22 (pre-PTM cells), C26 (late epithelial cells), C18 (rete cells) and C15 (SC) as potential ending cells. Numbering of clusters is according to Fig. S11B. As previously described the male gonadal somatic cells from the current dataset were mapped on top of the reference atlas of male gonad somatic cells using the FindTransferAnchors and MapQuery functions with default settings.

Supplementary References

Aliye, N., Fabbretti, A., Lupidi, G., Tsekoa, T. and Spurio, R. (2015). Engineering color variants of green fluorescent protein (GFP) for thermostability, pH-sensitivity, and improved folding kinetics. *Appl. Microbiol. Biotechnol.* **99**, 1205-1216. doi:10.1007/s00253-014-5975-1.

Anamthathmakula, P., Miryala, C.S.J., Moreci, R.S., Kyathanahalli, C., Hassan, S.S., Condon, J.C. and Jeyasuria, P. (2019). Steroidogenic Factor 1 (Nr5a1) is Required for Sertoli Cell Survival Post Sex Determination. *Sci. Rep.* **9**, 4452. doi:10.1038/s41598-019-41051-1

Baba, T, Otake, H, Sato, T, Miyabayashi, K, Shishido, Y, Wang, CY, Shima, Y, Kimura, H, Yagi, M, Ishihara, Y et al. (2014). Glycolytic genes are targets of the nuclear receptor Ad4BP/SF-1. *Nat. Commun.* **5**, 3634. doi:10.1038/ncomms4634.

Best, D., Sahlender, D.A., Walther, N., Peden, A.A. and Adams, I.R. (2008). Sdmg1 is a conserved transmembrane protein associated with germ cell sex determination and germline-soma interactions in mice. *Development* **135**, 1415-1425. doi:10.1242/dev.019497.

Chalmel, F. and Primig, M. (2008). The Annotation, Mapping, Expression and Network (AMEN) suite of tools for molecular systems biology. *BMC Bioinformatics* **9**, 86. doi:10.1186/1471-2105-9-86.

Farley, F.W., Soriano, P., Steffen, L.S. and Dymecki, S.M. (2000). Widespread recombinase expression using FLPeR (flipper) mice. *Genesis* **28**, 106-110. PMID: 11105051

Hao, Y., Hao, S., Andersen-Nissen, E., Mauck, W M.3rd, Zheng, S., Butler, A., Lee, M.J., Wilk, A.J., Darby, C., Zager, M., et al. (2021). Satija, Integrated analysis of multimodal single-cell data. *Cell* **184**, 3573-3587. doi:10.1016/j.cell.2021.04.048.

Ikeda, Y., Tagami, A., Maekawa, M. and Nagai, A. (2021). The conditional deletion of steroidogenic factor 1 (Nr5a1) in Sox9-Cre mice compromises testis differentiation. *Sci. Rep.* **11**, 4486. doi:10.1038/s41598-021-84095-y

Jonkers, J., Meuwissen, R., van der Gulden, H., Peterse, H., van der Valk, M. and Berns, A. (2001). Synergistic tumor suppressor activity of BRCA2 and p53 in a conditional mouse model for breast cancer. *Nat. Genet.* **29**, 418-425. doi:10.1038/ng747.

Lécureuil, C., Fontaine, I., Crepieux, P. and Guillou, F. (2002). Sertoli and granulosa cell-specific Cre recombinase activity in transgenic mice. *Genesis* **33**, 114-118. doi:10.1002/gene.10100.

Mayère, C., Regard, V., Perea-Gomez, A., Bunce, C., Neirijnck, Y., Djari, C., Bellido-Carreras, N., Sararols, P., Reeves, R., Greenaway, S., et al. (2022). Origin, specification and differentiation of a rare supporting-like lineage in the developing mouse gonad. *Sci. Adv.* 8, eabm0972. doi:10.1126/sciadv.abm0972.

McCarthy, D.J., Campbell, K.R., Lun, A.T. and Wills, Q.F. (2017). Scater: pre-processing, quality control, normalization and visualization of single-cell RNA-seq data in R. *Bioinformatics* **33**, 1179-1186. doi:10.1093/bioinformatics/btw77.

McGinnis, C.S., Murrow, L.M. and Gartner, Z.J. (2019). DoubletFinder: Doublet Detection in Single-Cell RNA Sequencing Data Using Artificial Nearest Neighbors. *Cell Syst.* **8**, 329-337. doi:10.1016/j.cels.2019.03.003.

Schindelin, J., Arganda-Carreras, I., Frise, E., Kaynig, V., Longair, M., Pietzsch, T. and Cardona, A. (2012). Fiji: an open-source platform for biological-image analysis. *Nat. Methods* **9**, 676-682. doi:10.1038/nmeth.2019

Srinivas, S., Watanabe, T., Lin, C.S., William, C.M., Tanabe, Y., Jessell, T.M. and Costantini, F. (2001). Cre reporter strains produced by targeted insertion of EYFP and ECFP into the ROSA26 locus. *BMC Dev. Biol.* **1**, 4. doi:10.1186/1471-213x-1-4.

Street, K., Risso, D., Fletcher, R.B., Das, D., Ngai, J., Yosef, N., Purdom, E. and Dudoit, S. (2018). Slingshot: cell lineage and pseudotime inference for single-cell transcriptomics. *BMC Genomics* **19**, 477. doi:10.1186/s12864-018-4772-0. doi:10.1186/s12864-018-4772-0.

Wang, Y.Q., Cheng, J.M., Wen, Q., Tang, J.X., Li, J., Chen, S.R. and Liu, Y.X. (2020). An exploration of the role of Sertoli cells on fetal testis development using cell ablation strategy. *Mol. Reprod. Dev.* **87**, 223-230. doi:10.1002/mrd.23309

Development • Supplementary information
