# Supplementary material for: Loss of NR5A1 in mouse Sertoli cells after sex determination changes cellular identity and induces cell death by anoikis
Source: Development. 2023 Dec 11;150(24):dev201710. doi: 10.1242/dev.201710 (PMC10753587; doi:10.1242/dev.201710)
Supplement: Supplementary file 1 [file develop-150-201710_review_history.pdf]

Loss of NR5A1 in mouse Sertoli cells after sex determination changes cellular identity and induces cell-death by anoikis

Sirine Souali-Crespo, Diana Condrea, Nadège Vernet, Betty Féret, Muriel Klopfenstein, Erwan Grandgirard, Violaine Alunni, Marie Cerciat, Matthieu Jung, Chloé Mayere, Serge Nef, Manuel Mark, Frédéric Chalmel and Norbert B. Ghyselinck
DOI: 10.1242/dev.201710

Editor: Patrick Tam

Review timeline

Original submission:	17 February 2023
Editorial decision:	6 April 2023
First revision received:	16 October 2023
Accepted:	9 November 2023

Original submission

First decision letter

MS ID#: DEVELOP/2023/201710

MS TITLE: Loss of NR5A1 in Sertoli cells after sex determination changes their cellular identity and induces their death by anoikis

AUTHORS: Sirine Souali-Crespo, Diana Condrea, Nadege Vernet, Betty Feret, Muriel Klopfenstein, Erwan Grandgirard, Violaine Alunni, Marie Cerciat, Matthieu Jung, Chloe Mayere, Serge Nef, Manuel P Mark, Frederic Chalmel, and Norbert B. Ghyselinck

I have now received all the referees' reports on the above manuscript. The referees' comments are appended below, or you can access them online: please go to BenchPress and click on the 'Manuscripts with Decisions' queue in the Author Area.

As you will see, the referees express interest in your work, but have some significant criticisms and recommend a substantial revision of your manuscript before we can consider publication. If you are able to revise the manuscript along the lines suggested (see also Editor's Note), which may involve further experiments, I will be happy receive a revised version of the manuscript. Your revised paper will be re-reviewed by one or more of the original referees, and acceptance of your manuscript will depend on your addressing satisfactorily the reviewers' major concerns. Please also note that Development will normally permit only one round of major revision.

If it would be helpful, you are welcome to contact us to discuss your revision in greater detail. Please send us a point-by-point response indicating your plans for addressing the referee's comments, and we will look over this and provide further guidance.

Please attend to all of the reviewers' comments and the Editor's note and ensure that you clearly highlight all changes made in the revised manuscript. Please avoid using 'Tracked changes' in Word files as these are lost in PDF conversion. I should be grateful if you would also provide a point-by-point response detailing how you have dealt with the points raised by the reviewers in the 'Response to Reviewers' box. If you do not agree with any of their criticisms or suggestions please explain clearly why this is so.

© 2023. Published by The Company of Biologists under the terms of the Creative Commons Attribution License (https://creativecommons.org/licenses/by/4.0/).

Reviewer 1

Advance summary and potential significance to field

This is the study of the phenotype analysis of the Plekha5Tg(AMH-cre)1; Nr5a1-cKO testes, in order to make clear the Nr5a1 roles in the SC from E 13.5 onwards. By using immunohistochemical , qRT-PCR and scRNA seq analyses, the authors demonstrated that Nr5a1-null SC impairs the expression of genes characteristic of SC identity such as Sox9 and Amh, together with altered landscape of adhesion and extracellular matrix-related genes, leading to their trans-differentiation into a SOX9/Foxl2-double negative (pre-granulosa cell-like) cells prior to the cell death by a Trp53-independent mechanism. The loss of SCs from E14.5-15.5 testes results in the defects in various other gonadal and extragonadal cells such as LC GC and internal and external genitalia. These findings indicate that the crucial roles of NR5A1 in the SCs after the period of sex determination safeguards cell identity for proper testicular development.

In contrast, the phenotypes of Nr5a1 cKO using a Sox9-cre or another Amh-cre line were already published previously. The defects in other gonadal and extragonadal cell types by the SC ablation was also previously shown by the in vivo cell depletion system using diphtheria toxin, etc. Thus, the novelty and significance of this study are limited into the scRNA seq data in the trans-differentiation of Nr5a1-null SCs.

Such transcriptome data in control and Nr5a1-null SCs will be of interest in the field; however, the scRNA seq data appear to be underutilized. There still remains unclear how a NR5A1 loss causes the cell death via a ‘pre-granulosa cell-like’ state.

Comments for the author

Dr. Souali-Crespo and his colleagues examined the detailed phenotypes of the Nr5a1-null Sertoli cells by using Amh-cre mice from E13.5 and onward. They revealed the trans-differentiation of the Nr5a1-null SCs into the SOX9/FOXl2-double negative pre-granulosa-like cells, together with altered gene expression ECM and adhesion. These transcriptomic alterations in the Nr5a1-null SCs may cause the rapid cell death by the a Trp53-independent manner. Such a SC loss after E14.5 causes the disorganized testis cords and affects the cell identity of several other gonadal cells and extra-gonadal tissues.

Indeed, this is the first report of the Nr5a1-cKO in the Sertoli cells at E13.5, but the phenotype reports using Nr5a1-null SCs (at E11.5 by Sox9-cre; and at E14.5 by another Amh-cre line), in addition to the phenotypic data of the fetal testes with Sertoli cell ablation (by using diphtheria toxin system), were already published in several previous studies. Therefore, the single cell level transcriptome in Nr5a1-null SCs is novel and interesting to this field. Therefore, the authors must carefully describe the scRNA seq data of Nr5a1-null pre-granulosa-like cells prior to the cell death.

Major comments

1) Cell autonomous or non-cell autonomous defects in Nr5a1-null SCs: A loss of NR5A1 (SF1/Ad4BP) actions cause the defective proliferation and cell loss of gonadal and adrenal progenitors as seen in the Nr5a1-null embryos. It was previously shown that NR5A1 (SF1/Ad4BP) action promotes the production of the energy carriers ATP and nicotinamide adenine dinucleotide phosphate together with increased expression of genes involved in glucose metabolism (Nat Commun 2014; doi: 10.1038/ncomms4634). The authors should pick up and discuss the metabolic defects in Nr5a1-null SCs at the base of the scRNA seq data.

2) Trp53-independent cell death by anoikis: There still remains obscure whether or not anoikis occurs in in the Nr5a1-null SCs. Is it possible to provide the additional experimental evidence in this point; for example, an in vitro rescue experiment using exogenous ECM supply?.

3) Testis specific vasculature may be crucial for the glucose/oxygen supply to the fetal Sertoli cells inside the testis cords. Such testis-specific vascularization is also regulated by paracrine factors derived from the Sertoli cells in developing testes. In the Nr5a1-null SC testis, the vascular structure is normally maintained? This is because the maintenance of the SCs that requires the high glucose energy for the ECM production (Matoba et al.,2008; doi: 10.1016/j.ydbio.2008.09.004).

© 2023. Published by The Company of Biologists under the terms of the Creative Commons Attribution License (https://creativecommons.org/licenses/by/4.0/).

Specific comments

1) P2. L5-6 in Abstract: '…… die by a Trp53-independent mechanism.' 'Trp53-independent' is not informative. This cell death is apoptosis, isn't it? Because the present data provide not only the positive signals for TUNEL staining (Fig. 3F), but also several GO terms associated with apoptotic cell death in the SC clusters (Table S3).

2) P3. Last sentence in Introduction: '….. transdifferentiate into female somatic cells' Prior to cell death, SOX9/FOXL2-double negative cells are not female somatic cells, maybe intersex that falls outside the strict male/female binary somatic cells.

3) P4, last paragraph & Fig. 3A-D. In the Nr5a1 cKO testes, YFP+ SC cells completely disappear by 16.5?
If a few YFP-positive cells can survive, are they located near the mesonephric side (i.e., some YFP+ SCs near the rete testis)?

4) P7. L12; Fig. S6-B: 'It ended at the junction with C21 with rete testis cells, originating exclusively from the Nr5a1SC-/- testes, and in which cre was active (Nr5a1-/Yfp+/Pax8+/Aldh1a3+ cells) (Suppl. Fig. S6B).
Accordingly, IHC experiments confirmed that rete testis cells actually expressed NR5A1, but not YFP control and Nr5a1SC-/- testes (Suppl. Fig. S6, C to J).'
A Nr5a1-/Yfp+/Pax8+/Aldh1a3+ subcluster (green rectangles in Fig. S6-B) indicates the presence of a certain subpopulation of Nr5a1-cKO (Nr5a1-/Yfp+) rete testis cells in control testes (green rectangle). Is it correct? What is the control mouse genotype used in scRNA seq? Because Yfp- SC cells in C19 are derived from the Yfp- control testes. This Nr5a1-cKO (Nr5a1-/Yfp+) rete testis subcluster was produced by Amh-cre activity?

5) P8, 'Nonetheless, the process of trans-differentiation was not fully achieved because IHC experiments failed to evidence expression of FOXL2 in the Nr5a1SC-/- mutant testis at E15.5.' This is a speculative sentence, because the transcriptional steps for the trans-differentiation into the FOXL2 induction pathway may be unclear at present.

6) P9, 2nd paragraph in discussion and Fig. 8: The authors must carefully display the scRNA seq data and their interpretation in this paragraph and figure 8. For examples, the authors mentioned the upregulation of Wnt4 and Rspo1, but both Fgf9 and Fgfr2 antagonistic signals for Wnt4/Rspo1 was also up-regulated in the Nr5a1-null SCs. Together with increased expression of a stromal marker, NR2f2, they may be a SOX9/Foxl2-double negative intersex stromal cells.

7) Table S3: The up-regulated genes in Nr5a1-null SCs revealed the GO term associated with apoptotic cell death (e.g., 'mitochondrial fragmentation involved in apoptotic process, regulation of mitochondrial membrane permeability involved in apoptotic process), together with metabolic alterations of ATP synthesis(e.g., 'ATP metabolic process', 'proton motive force-driven mitochondrial ATP synthesis'). NR5A1 (SF1/Ad4BP) can act to produce the energy carriers ATP and nicotinamide adenine dinucleotide phosphate, together with increased expression of genes involved in glucose metabolism (Nat Commun 2014; doi: 10.1038/ncomms4634). Such crucial information is missing in the discussion.

8) P9, bottom; 'the last sentence in this paragraph: In addition, SC die by distinct mechanisms depending on the stage at which NR5A1 is lost.'
This sentence is unclear. Maybe rewrite.

9) p.10, upper paragraph: This para is the important conclusion of the potential mechanism on the cell death of Nr5a1-null SCs by anoikis. The anoikis causes apoptosis cell death or not? ScRNA seq data in SCs showed several GO terms associated with apoptotic cell death.

10) P.10. L5-6 from bottom: 'Actually, CYP26B1 is required in SC to prevent meiotic initiation and to maintain GC in an undifferentiated state (48). 'FGF9 is also up-regulated in Nr5a1-null SCs, which may suppress meiosis and promotes male germ cell fate in mice (doi: 10.1016/j.devcel.2010.08.010).

© 2023. Published by The Company of Biologists under the terms of the Creative Commons Attribution License (https://creativecommons.org/licenses/by/4.0/).

11) P.12, 'Mice' in M&M: The sentence of 'Plekha5Tg(AMH-cre)1Flor;Nr5a1+/+; Gt(ROSA)26Sortm1(EYFP)Cos and Plekha5Tg(AMH-cre)1Flor;Nr5a1L2/L2; Gt(ROSA)26Sortm1(EYFP)Cos males are referred to as control and Nr5a1SC-/- mutant fetuses, respectively.' must be added.

Reviewer 2

Advance summary and potential significance to field

In this manuscript the authors attempt to tease out the functional role of NR5A1 in early Sertoli cell (SC) development and its subsequent impact on testis development. In an elegant series of experiments, they ablated Nr5a1 in SCs from E13.5 onwards (after testis determination has occurred) by creating Nr5a1SC-/- mice. Loss of Nr5a1 at this time point decreased the expression of known SC genes such as Sox9, Amh, Sox8 and Sox10. Intriguingly, they showed that SCs died by E14.5 by a Trp53-independent mechanism. Furthermore, loss of Nr5a1 induced disorganised cords due to loss of clear membrane boundaries and germ cells inappropriately entered meiosis and subsequently died. Single cell RNA transcriptomics suggest that Nr5a1SC-/- SCs become partially granulosa-like based on ovarian markers. Finally, they show that Nr5a1SC-/- adult males display Mullerian duct derivatives resulting in a mixture of male and female internal reproductive organs.

In the Nr5a1SC-/- mice the adhesion and extracellular matrix genes show altered expression patterns. This potential disruption to the extracellular matrix may result in cell detachment and thus death by a Trp53- independent mechanism such as anoikis. While not definitively demonstrated there is strong circumstantial evidence to support this claim. Overall, I found the experiments were well designed, the results and figures very clear. Consequently, the conclusions drawn were strongly supported by clear and compelling data. This work significantly extends our understanding of the role of Nr5a1 in SC development and its impact on testis development.

Comments for the author

Required Changes
1)I found the discussion wanting. Specifically, I would like to see the discussion comparing the results of this study to many other similar studies in mice such as Anamthathmakula et al (2019) Steroidogenic Factor 1 (Nr5a1) is required for Sertoli cell survival post sex determination. Scientific Reports. 9: 4452 and other similar papers. Particularly, how these studies relate to their conclusion that NR5A1 in SC (after sex determination) safeguards cell identity, maintains proper cord structure, prevents germ cells entering meiosis and allows adult Leydig cells to emerge.

2)Finally, Figure 8 Summary of the alterations induced by the loss of NR5A1 in Sertoli cells after sex determination is an excellent figure that draws together all the manuscripts findings. However, I think the Figure legend is incorrect in saying: blue and yellow ovals are up- and down-regulated genes, respectively. I think the authors mean the reverse. ie. Yellow is up-regulated, and Blue is down-regulated – clearly this is important to rectify.

Reviewer 3

Advance summary and potential significance to field

This manuscript describes the consequences of murine Sertoli Cells (SC) lacking NR5A1 from E13.5. These cells transiently transdifferentiate to acquire a pre-granulosa cell-like identity, but also show modified expressed of adhesion molecules and ECM and die by a TRP53-independent mechanism. This data, together with other studies, suggest that there specific windows in early Sertoli cell development when they show plasticity for changes in cell identity.

Comments for the author

General comments

© 2023. Published by The Company of Biologists under the terms of the Creative Commons Attribution License (https://creativecommons.org/licenses/by/4.0/).

One of the main issues of this manuscript is the English style and grammar. As a result, it is a very difficult paper to read. Almost every sentence in the manuscript requires correction. It is recommended that the authors spend some time with a native English speaker to correct the style.

The other major issue is the construction of the results and the discussion sections. In particular, the discussion alludes to data that is not mentioned in the results section (examples below). This adds to the difficulty in reading this text and as a consequence it is also difficult for the non-specialist reader – who is not familiar with sex-determination – to grasp the importance of this work.

The authors should rewrite the manuscript, especially the results and dicussion, taking into consideration the English style, the accessibility of the manuscript for non-specialist readers and also to include in the results section, the data that is mentioned in the results.

Specific comments

The data and analysis are based on an NR5A1 AMH-cre construct. This is not ideal as it requires the expression of AMH from Sertoli cells and essentially one is observing the effects of ablation of NR5A1 in mate sex-determination – after the expression of SRY, SOX9 and DMRT1. Surprisingly the authors observed YFP at day 12.5 in SC – can the authors explain why excision occurred so early? Since NR5A1 directly controls the expression of AMH – will this compromise CRE-excision?

The authors report that ablation of NR5A1 resulted in the downregulation of the known suspects in testis-determination – SOX8, SOX9 etc… Howeve,r the consequences of the ablation of NR5A1 on the expression of the Sertoli Cell factor DMRT1 are not reported in the results section. DMRT1 is a key player in maintaining the Sertoli cell identity in mice (e.g. Lindeman et al., Nucleic Acids Res. 2021; 49:6144-6164; Matson et al., Nature. 2011;476:101-4).The precise role of DMRT1 in early murine testis formation is also debated. In their model, the authors allude to a loss of expression of DMRT1 but this is mention only in the discussion (page 9). Since DMRT1 is such an important factor in the process, the authors should describe in detail the changes in expression in DMRT1 – together with a supporting figure.

Page 9 – the discussion of the effects of NR5A1 ablation at specific time points on cellular plasticity could be rewritten possibility with a supporting supplementary figure.

First revision

<u>Author response to reviewers' comments</u>

<u>**Editor's Note:**</u>

> *"Please highlight the novelty of the findings of this mutant mouse model on the impact of Sertoli cell deficiency on testis development in relation to those of other studies of Sertoli cell ablation at different developmental time points, and provide an in-depth analysis of the transcriptomic data to support the inference of cellular viability, aberrant transition of cell states and disruption of tissue morphogenesis in the testis."*

Dear editor, we have deepened our analysis of the scRNA-seq data, by notably looking at the differentiation status of the NR5A1-deficient Sertoli cells (SC) and at their metabolism, and we have also performed additional experiments to support the idea that SC die by anoikis. In the discussion section, we have better compared our model to the other ones already published (ablation with *Sox9*-Cre, another *Amh*-Cre, or by genetic ablation using Diphtheria toxin expression), underlining the differences and the similarities.

<u>**Reviewer #1 Comments for the Author:**</u>

© 2023. Published by The Company of Biologists under the terms of the Creative Commons Attribution License (https://creativecommons.org/licenses/by/4.0/).

"[…] This is the first report of the Nr5a1-cKO in the Sertoli cells at E13.5, but the phenotype reports using Nr5a1-null SCs (at E11.5 by Sox9-cre; and at E14.5 by another Amh-cre line), in addition to the phenotypic data of the fetal testes with Sertoli cell ablation (by using diphtheria toxin system), were already published in several previous studies. Therefore, the single cell level transcriptome in Nr5a1-null SCs is novel and interesting to this field. Therefore, the authors must carefully describe the scRNA seq data of Nr5a1-null pre- granulosa-like cells prior to the cell death."

We thank Reviewer #1 for his/her careful analysis of our MS. Considering his/her comments we have introduced several new findings and better discussed most of them, as indicated above.

Major comments

1) *"It was previously shown that NR5A1 (SF1/Ad4BP) action promotes the production of the energy carriers ATP and nicotinamide adenine dinucleotide phosphate, together with increased expression of genes involved in glucose metabolism (Nat Commun 2014; doi: 10.1038/ncomms4634). The authors should pick up and discuss the metabolic defects in Nr5a1-null SCs at the base of the scRNA seq data."*

In the revised version of our MS we have added results about deregulated gene expression according to the scRNA- seq data (Fig. S13A), production of ATP using FACS-purified control and mutant SC (Fig. S13B), and glycolysis (Fig. S13C,D). We show that the expression of most the genes involved in ATP synthesis are upregulated in NR5A1- deficient SC but ATP production is not significantly changed. This is a surprising data, given the changes observed in gene expression. However, it likely reflects the fact that the mutant SC are dying and that mitochondria are consequently altered. It is also possible that NR5A1 does not control energy metabolism the same way in SC and in adrenocortical Y-1 cells (Baba et al., 2014). Regarding the glycolysis pathways, we show that some genes are upregulated, but some others are downregulated. Amongst the latter, we have focused on the expression of *Hk1*, *Aldoa*, *Pgam1* and *Eno1* which all contain a NR5A1-response element in their gene promoter (Baba et al., 2014). Their decreased expression may be a direct consequence of the loss of NR5A1. Given (i) the odd result obtained for ATP production, (ii) the fact that NR5A1-deficient SC are dying by nature, (iii) the difficulty to FACS-purify SC from fetal gonads, and (iv) the 3R's rule that requires the number of animals used to be kept to a minimum, we did not measure glucose uptake/metabolism.

The changes we introduced on this matter are page 12, lines 302-321 (Results section); page 22, lines 578-583 (Math & Meth section) in the revised MS, as well as Fig. S13A-D in Supplementary Information.

2) *"There still remains obscure whether or not anoikis occurs in in the Nr5a1-null SCs. Is it possible to provide the additional experimental evidence in this point; for example, an in vitro rescue experiment using exogenous ECM supply?".*

We agree with the reviewer. Experiments were missing to support our proposal that NR5A1-deficient SC die by anoikis. The idea of rescuing the phenotype by using exogenous ECM as published elsewhere (Matoba et al., 2008 *Dev. Biol*. 324, 76-87) is appealing, but its implementation looks highly complicated. In the present case the ECM is not simply lost (as it is the case in the reference cited above), but it is profoundly changed: a dozen of male-type ECM components is exchanged by female-type ECM components in the mutant SC (Table S3). Embedding and incubating *in vitro* mutant gonads into a matrix Matrigel (BD Biosciences) containing 60% laminin and 30% collagen IV may not be sufficient to change an existing, but inappropriate matrix, at the basement membrane of SC.

Thus, to meet the reviewer's comment, we have better defined anoikis and explained how it can be evidenced, looked at the expression of anoikis-related genes (Fig; 7B), quantified caspase 3 activity in FACS-purified control and NR5A1-deficient SC (Fig. 7C), show that SC viability *in vitro* is dependent upon their attachment (Fig. 7D-H), and discussed the results. The latter experiment was performed using the MSC-1 SC line as an *in vitro* surrogate model of control SC. We chose this approach because each measurement of cell viability by MTT assay or by calcein AM/ethD-1 assay requires 30,000 cells per well. As one pair of E13.5 gonads allows purification of only 5,000 SC by

© 2023. Published by The Company of Biologists under the terms of the Creative Commons Attribution License (https://creativecommons.org/licenses/by/4.0/).

FACS, obtaining 30,000 SC by FACS purification would have required 6 fetuses for 1 measurement. Repeating at least 2 times each of the two experiments with triplicates would have required a total of 72 fetuses. This was against the 3R's rule.

In addition, the paper mentioned by the reviewer (Matoba et al., 2008 *Dev. Biol*. 324, 76-87) is now cited in the revised version of the MS (page 14, line 369).

The changes we introduced on this matter are page 6, lines 136-138 (Results section); page 14, lines 366-391 (Discussion section); page 22, lines 564-577 (Math & Meth section), and page 34, lines 933-945 (Fig Legends) and Fig. 7A-H in the revised MS.

> 3) "*Testis specific vasculature may be crucial for the glucose/oxygen supply to the fetal Sertoli cells inside the testis cords. Such testis-specific vascularization is also regulated by paracrine factors derived from the Sertoli cells in developing testes. In the Nr5a1-null SC testis, the vascular structure is normally maintained? This is because the maintenance of the SCs that requires the high glucose energy for the ECM production (Matoba et al.,2008; doi: 10.1016/j.ydbio.2008.09.004).*"

To meet the reviewer's comment, we have analyzed the vasculature in control and mutant testes at E13.5 and E14.5 by immunohistochemistry with antibodies against YPF to visualize SC and PECAM to visualize blood vessels. The samples have been imaged using a confocal spinning disk microscope or by light-sheet microscopy. The data show that vasculature is not altered. Regarding glucose energy for ECM production, the published data show that it is important during the early stage of pre-Sertoli cell differentiation by SOX9 (i.e., at E11.5 and for 24-48 hours using *in vitro* cultured gonads). In the present study, we deleted NR5A1, and consequently lost SOX9 expression, from E13.5-E14.5, which is 2 days later. Therefore, it is unlikely that glucose production is involved the phenotype displayed by the *Nr5a1*$^{SC-/-}$ mutants we describe in our MS. Consistent with our understanding, page 86 in Matoba et al., 2008 (*Dev. Biol*. 324, 76-87) it is indicated that "*After 12.0 dpc, the maintenance mechanism for the high levels of SOX9 expression appears to become stabilized in the differentiating Sertoli cells because, in organ cultures using 12.0-dpc XY genital ridges, both SOX9 expression and cord formation are properly maintained in most XY glucose depleted explants for 2-3 days*".

The changes we introduced on this matter are page 9, lines 227-254 (Results section); page 19, lines 501-503 (Math & Meth section) in the revised MS, as well as Fig. S2A-F in Supplementary Information.

Specific comments

> 1) "*P2. L5-6 in Abstract: 'Trp53-independent' is not informative. This cell death is apoptosis, isn't it? Because the present data provide not only the positive signals for TUNEL staining (Fig. 3F), but also several GO terms associated with apoptotic cell death in the SC clusters (Table S3).*"

We have clarified the definition of anoikis, which is, indeed, a special case of apoptosis that do ne require the involvement of TRP53. The changes we introduced about anoikis are page 6, lines 136-138 (Results section); page 14, lines 366-391 (Discussion section); page 22, lines 564-577 (Math & Meth section), and page 34, lines 933-945 (Fig Legends) and Fig. 7A-H in the revised MS.

The changes we introduced about anoikis are page 6, lines 136-138 (Results section); page 14, lines 366-391 (Discussion section); page 22, lines 564-577 (Math & Meth section), and page 34, lines 933-945 (Fig Legends) and Fig. 7A-H in the revised MS.

> 2) "*P3. Last sentence in Introduction: '….. transdifferentiate into female somatic cells' Prior to cell death, SOX9/FOXL2-double negative cells are not female somatic cells, maybe intersex that falls outside the strict male/female binary somatic cells.*"

The reviewer is right. We have included this notion, notably by adding results comparing our data set to the male somatic cells of the gonad atlas published recently (Mayère et al., 2022). We actually

© 2023. Published by The Company of Biologists under the terms of the Creative Commons Attribution License (https://creativecommons.org/licenses/by/4.0/).

found that the Nr5A1-deficient SC acquire four cellular identities that can be distinguished at the molecular level: male-oriented supporting progenitors-like, intersex supporting progenitors-like, female-oriented supporting progenitors-like, and pre- granulosa-like.

The changes we introduced on that matter are page 2, lines 45-49 (Abstract), page 11, lines 277-301 (Results section); page 13, lines 334-365 (Discussion section), page 20, lines 509-521 (Math & Meth section), and page 35, lines 966-989 (Fig Legends), Fig; 9 in the revised MS, as well as Fig. S10 to S12 in Supplementary Information.

3) *"P4, last paragraph & Fig. 3A-D. In the Nr5a1 cKO testes, YFP+ SC cells completely disappear by 16.5? If a few YFP-positive cells can survive, are they located near the mesonephric side (i.e., some YFP+ SCs near the rete testis)?"*

We did not look whether some SC are still present at E16.5 in *Nr5a1*$^{SC-/-}$ testes. We have nonetheless analyzed newborn (Fig. S3A), and postnatal day 15 or 60 testes (Fig. 4N-S), in which SC were totally lost. We have evidence that SC surviving at E15.5 are indeed located on the mesonephric side of the gonad.

The changes we introduced on that matter are in Supplementary Information (Fig. S14).

4) *"P7. L12; Fig. S6-B: '[…]'A Nr5a1-/Yfp+/Pax8+/Aldh1a3+ subcluster (green rectangles in Fig. S6-B) indicates the presence of a certain subpopulation of Nr5a1-cKO (Nr5a1-/Yfp+) rete testis cells in control testes (green rectangle). Is it correct? What is the control mouse genotype used in scRNA seq? Because Yfp- SC cells in C19 are derived from the Yfp- control testes. This Nr5a1-cKO (Nr5a1-/Yfp+) rete testis subcluster was produced by Amh-cre activity?"*

We sincerely apologize for the mistakes that were introduced into the previous version of Fig. S6. They are likely at the origin of the misunderstanding. As a matter of fact, circles stand for cells from the control testis, while triangles stand for cells from the *Nr5a1*$^{SC-/-}$ testis. In addition, green stands for *Nr5a1*$^{-}$/*Yfp*$^{+}$/*Pax8*$^{-}$/*Aldh1a3*$^{-}$ cells. On top of that, it was not clearly specified that the control gonads used for the scRNA-seq experiment were from fetuses lacking the YFP transgene, while those used for the IHC experiment illustrated in panels C-J were from fetuses carrying the YFP-transgene. We carefully verified all the labeling on the revised version of our MS.

The changes we introduced on that matter are page 9, lines 219-226 (Results section); page 18, lines 473-476 and lines 556-557 (Math & Meth section) in the revised MS, as well as Fig. S7 and its legend in Supplementary Information.

5) *"P8, Nonetheless, the process of trans-differentiation was not fully achieved because IHC experiments failed to evidence expression of FOXL2 in the Nr5a1SC-/- mutant testis at E15.5.'This is a speculative sentence, because the transcriptional steps for the trans-differentiation into the FOXL2 induction pathway may be unclear at present."*

We agree with the reviewer. We have modified this sentence (page 11, lines 274-275).

6) *"P9, 2nd paragraph in discussion and Fig. 8: The authors must carefully display the scRNA seq data and their interpretation in this paragraph and figure 8. For examples, the authors mentioned the upregulation of Wnt4 and Rspo1, but both Fgf9 and Fgfr2, antagonistic signals for Wnt4/Rspo1 was also up-regulated in the Nr5a1-null SCs. Together with increased expression of a stromal marker, NR2f2, they may be a SOX9/Foxl2- double negative intersex stromal cells."*

We thank the reviewer for his/her very interesting comment. We have actually deepened our analysis of the scRNA- seq data, and show that that the Nr5A1-deficient SC acquire four cellular identities that can be distinguished at the molecular level: male-oriented supporting progenitors-like, intersex supporting progenitors-like, female-oriented supporting progenitors-like, and pre-granulosa-like.

The changes we introduced on that matter are page 2, lines 45-49 (Abstract), page 11, lines 277-301 (Results section); page 13, lines 334-365 (Discussion section), page 20, lines 509-521 (Math &

© 2023. Published by The Company of Biologists under the terms of the Creative Commons Attribution License (https://creativecommons.org/licenses/by/4.0/).

Meth section), and page 35, lines 966-989 (Fig Legends), Fig; 9 in the revised MS, as well as Fig. S10 to S12 in Supplementary Information.

7) *"Table S3: The up-regulated genes in Nr5a1-null SCs revealed the GO term associated with apoptotic cell death (e.g., 'mitochondrial fragmentation involved in apoptotic process, regulation of mitochondrial membrane permeability involved in apoptotic process), together with metabolic alterations of ATP synthesis (e.g., 'ATP metabolic process', 'proton motive force-driven mitochondrial ATP synthesis'). NR5A1 (SF1/Ad4BP) can act to produce the energy carriers ATP and nicotinamide adenine dinucleotide phosphate, together with increased expression of genes involved in glucose metabolism (Nat Commun 2014; doi: 10.1038/ncomms4634). Such crucial information is missing in the discussion."*

As indicated above (major comments #1 and #2) we have better defined anoikis/apoptosis and explained how it can be evidenced, looked at the expression of anoikis-related genes (Fig; 7B), quantified caspase 3 activity in FACS-purified control and NR5A1-deficient SC (Fig. 7C), show that SC viability *in vitro* is dependent upon their attachment (Fig. 7D-H), and discussed the results. We have also added results about deregulated gene expression according to the scRNA-seq data (Fig. S13A), production of ATP using FACS-purified control and mutant SC (Fig. S13B), and glycolysis (Fig. S13C,D).

The changes we introduced on these two matters are page 6, lines 136-138 and page 12, lines 302-321 (Results section); page 14, lines 366-391 (Discussion section); page 22, lines 564-577 and page 22, lines 578-583 (Math & Meth section), and page 34, lines 933-945 (Fig Legends) and Fig. 7A-H in the revised MS, as well as Fig. S13A-D in Supplementary Information.

8) *"P9, bottom; 'the last sentence in this paragraph: In addition, SC die by distinct mechanisms, depending on the stage at which NR5A1 is lost.' This sentence is unclear. Maybe rewrite."*

We apologize for being not clear. We have modified the complete paragraph (page 15, lines 392-398).

9) *"P.10, upper paragraph: This para is the important conclusion of the potential mechanism on the cell death of Nr5a1-null SCs by anoikis. The anoikis causes apoptosis cell death or not? ScRNA seq data in SCs showed several GO terms associated with apoptotic cell death."*

As indicated above (major comment #2) we have better defined anoikis/apoptosis and explained how it can be evidenced, looked at the expression of anoikis-related genes (Fig; 7B), quantified caspase 3 activity in FACS- purified control and NR5A1-deficient SC (Fig. 7C), show that SC viability *in vitro* is dependent upon their attachment (Fig. 7D-H), and discussed the results.

The changes we introduced about anoikis are page 6, lines 136-138 (Results section); page 14, lines 366-391 (Discussion section); page 22, lines 564-577 (Math & Meth section), and page 34, lines 933-945 (Fig Legends) and Fig. 7A-H in the revised MS.

10) *"P.10. L5-6 from bottom: 'Actually, CYP26B1 is required in SC to prevent meiotic initiation and to maintain GC in an undifferentiated state (48). 'FGF9 is also up-regulated in Nr5a1-null SCs, which may suppress meiosis and promotes male germ cell fate in mice (doi: 10.1016/j.devcel.2010.08.010)."*

The reviewer is right, *Fgf9* and *Fgfr1* are upregulated in the NR5A1-deficient SC. Whether it could account for the initiation of meiosis in the *Nr5a1*$^{SC-/-}$ testes is now discussed (page 16, lines 424-432).

11) *"P.12, 'Mice' in M&M: The sentence of 'Plekha5Tg(AMH-cre)1Flor;Nr5a1+/+; Gt(ROSA)26Sortm1(EYFP)Cos and Plekha5Tg(AMH-cre)1Flor;Nr5a1L2/L2; Gt(ROSA)26Sortm1(EYFP)Cos males are referred to as control and Nr5a1SC-/- mutant fetuses, respectively.' must be added."*

This sentence is added in the revised MS (page 18, lines 473-476). In addition, the genotype of the

© 2023. Published by The Company of Biologists under the terms of the Creative Commons Attribution License (https://creativecommons.org/licenses/by/4.0/).

fetuses used for scRNA-seq experiment were added (page 21, lines 556-558).

Reviewer #2 Comments for the Author:

"In an elegant series of experiments, they ablated Nr5a1 in SCs from E13.5 onwards (after testis determination has occurred) by creating Nr5a1SC-/- mice. [...] In the Nr5a1SC-/- mice the adhesion and extracellular matrix genes show altered expression patterns. This potential disruption to the extracellular matrix may result in cell detachment and thus death by a Trp53- independent mechanism such as anoikis. While not definitively demonstrated there is strong circumstantial evidence to support this claim. Overall, I found the experiments were well designed, the results and figures very clear. Consequently, the conclusions drawn were strongly supported by clear and compelling data. This work significantly. extends our understanding of the role of Nr5a1 in SC development and its impact on testis development."

We sincerely thank the reviewer for his/her kind comments. The revised version of our MS now present evidence for death of NR5A1-deficient SC by anoikis.

Required Changes

1) *"I found the discussion wanting. Specifically, I would like to see the discussion comparing the results of this study to many other similar studies in mice such as Anamthathmakula et al (2019) Steroidogenic Factor 1 (Nr5a1) is required for Sertoli cell survival post sex determination. Scientific Reports. 9: 4452 and other similar papers. Particularly, how these studies relate to their conclusion that NR5A1 in SC (after sex determination) safeguards cell identity, maintains proper cord structure, prevents germ cells entering meiosis and allows adult Leydig cells to emerge."*

To meet the reviewer criticism, we have modified the discussion and added the comparison between our model and the published ones on several occasions: first when discussing the change of SC identity, second when discussing the death of SC by anoikis. We have also modified the discussion about meiotic entry in GC, and totally remodeled the section discussing the fate of cell types other than SC in the *Nr5a1*$^{SC-/-}$ testis, notably for Leydig cells and peritubular myoid cells. On top of that, we now discuss the identities that NR5A1-deficient SC adopt before dying by anoikis. We hope reviewer #2 will consider our changes as suitable.

More specifically, the changes we introduced are page 11, lines 277-301 (Results section); page 14, lines 355-365 and page 15, lines 392-398, as well as page 16-18, lines 424-458 (Discussion section) in the revised MS, as well as Table S5 in Supplementary information.

2) *"Finally, Figure 8 Summary of the alterations induced by the loss of NR5A1 in Sertoli cells after sex determination is an excellent figure that draws together all the manuscripts findings. However, I think the Figure legend is incorrect in saying: blue and yellow ovals are up- and down-regulated genes, respectively. I think the authors mean the reverse. ie. Yellow is up-regulated, and Blue is down-regulated – clearly this is important to rectify."*

We apologize for this mistake. The sentence is corrected in the revised MS (page 36, lines 991-992).

Reviewer #3 Comments for the Author: General comments

"One of the main issues of this manuscript is the English style and grammar. As a result, it is a very difficult paper to read. Almost every sentence in the manuscript requires correction. It is recommended that the authors spend some time with a native English speaker to correct the style."

We apologize for this. The revised version has been carefully read by a native English scientist (Dr Patrick REILLY). We hope the revised version reads easier.

© 2023. Published by The Company of Biologists under the terms of the Creative Commons Attribution License (https://creativecommons.org/licenses/by/4.0/).

> *"The other major issue is the construction of the results and the discussion sections. In particular, the discussion alludes to data that is not mentioned in the results section (examples below). This adds to the difficulty in reading this text and as a consequence it is also difficult for the non-specialist reader – who is not familiar with sex-determination – to grasp the importance of this work."*

> *"The authors should rewrite the manuscript, especially the results and discussion, taking into consideration the English style, the accessibility of the manuscript for non-specialist readers and also to include in the results section, the data that is mentioned in the results."*

We sincerely apologize for this also. The revised version is profoundly remodeled and the Discussion section is almost totally rewritten. We hope the results and their discussion are easier to read now.

Specific comments

> *"The data and analysis are based on an NR5A1 AMH-cre construct. This is not ideal as it requires the expression of AMH from Sertoli cells and essentially one is observing the effects of ablation of NR5A1 in mate sex-determination – after the expression of SRY, SOX9 and DMRT1. Surprisingly the authors observed YFP at day 12.5 in SC – can the authors explain why excision occurred so early? Since NR5A1 directly controls the expression of AMH – will this compromise CRE-excision?"*

The transgene we used (*Amh-Cre*) contains a 3504 bp-long fragment of the human AMH promoter, a cre recombinase gene, and genomic sequences from MT1 to provide mRNA secondary structure stability. The gene trap is inserted into intron 3 of the *Plekha5* locus. It recapitulates expression of Cre in the mouse foetal Sertoli cells (evidence at E15.5 in the original paper) and in post-natal granulosa cells (Lécureuil et al., 2002). Contrary to the comment of the reviewer, the transgene does not "*require expression of AMH from Sertoli cells*": AMH does not regulate expression of Cre. It is true that the transgene contains the NR5A1-response element described at -150 to -100 relative to the TSS in the human *Amh* promoter (de Santa-Barbara et al., 1998, *Mol. Cell Biol.* 18, 6653-6665). However, this does not call into question excision the of loxP-flanked alleles of *Nr5a1* by Cre, because NR5A1 is expressed and present in Sertoli cells before turning on AMH gene/Cre transgene expression. Indeed, our Fig. 1B clearly shows that NR5A1 is normally present in Sertoli cells at E12.5. It also shows that NR5A1 is clearly lost from all Sertoli cells at E13.5.

We noticed some excision of the reporter transgene *Rosa26-loxP-STOP-loxP-YFP* at E12.5 (but not all Sertoli cells). This is much earlier than anticipated from the original publication. It is admitted for years that Cre-mediated recombination frequency might vary between genomic target locations and the genetic background (Vooijs et al. 2001, *EMBO Rep.* 2, 292-297). Our mice are on a mixed 129Sv/C57BL6 background, not a pure one as in Lécureuil et al., 2002. Nonetheless, NR5A1 is still present in all Sertoli cells at E12.5 (Fig. 1B). Then there is no doubt on excision of *Nr5a1* between E12.5 and E13.5, because NR5A1 is totally lost at E13.5 (Fig. 1D). We have analysed several gonads, from several mutants, all of them showing the same result.

> *"[…] the consequences of the ablation of NR5A1 on the expression of the Sertoli Cell factor DMRT1 are not reported in the results section. DMRT1 is a key player in maintaining the Sertoli cell identity in mice (e.g. Lindeman et al., Nucleic Acids Res. 2021; 49:6144-6164; Matson et al., Nature. 2011;476:101-4).The precise role of DMRT1 in early murine testis formation is also debated. In their model, the authors allude to a loss of expression of DMRT1 but this is mention only in the discussion (page 9). Since DMRT1 is such an important factor in the process, the authors should describe in detail the changes in expression in DMRT1 – together with a supporting figure."*

The reviewer is right, Dmrt1 was only quickly mentioned in the discussion. To meet its criticism, we have described its expression in the Result section, and discussed its possible involvement in the Discussion section.

More specifically, the changes we introduced are page 9, lines 216-217 (Results section); page 13, lines 328-332 (Discussion section) and Fig. 6M in the revised MS.

© 2023. Published by The Company of Biologists under the terms of the Creative Commons Attribution License (https://creativecommons.org/licenses/by/4.0/).

"Page 9 – the discussion of the effects of NR5A1 ablation at specific time points on cellular plasticity could be rewritten possibility with a supporting supplementary figure."

To meet the reviewer criticism, we have modified the discussion and added the comparison between our model and the published ones on several occasions: first when discussing the change of SC identity, second when discussing the death of SC by anoikis. We hope reviewer #3 will consider our changes as suitable.

More specifically, the changes we introduced are page 14, lines 355-365 and page 15, lines 392-398 (Discussion section) in the revised MS, as well as Table S5 in Supplementary information.

Second decision letter

MS ID#: DEVELOP/2023/201710

MS TITLE: Loss of NR5A1 in mouse Sertoli cells after sex determination changes cellular identity and induces cell-death by anoikis

AUTHORS: Sirine Souali-Crespo, Diana Condrea, Nadege Vernet, Betty Feret, Muriel Klopfenstein, Erwan Grandgirard, Violaine Alunni, Marie Cerciat, Matthieu Jung, Chloe Mayere, Serge Nef, Manuel P Mark, Frederic Chalmel, and Norbert B. Ghyselinck
ARTICLE TYPE: Research Article

I am happy to tell you that your manuscript has been accepted for publication in Development, pending our standard ethics checks. (please also see Editor's note.)

Reviewer 1

Advance summary and potential significance to field

This is the first report showing the single cell level transcriptome in Nr5a1-null Sertoli cells at E13.5-14.5 altering differentiation and metacolic status prior to cell death by anoikis.

Comments for the author

The authors have revised the manuscript properly and carefully. This reviewer has no futher comment.

Reviewer 2

Advance summary and potential significance to field

The authors have satisfactorily addressed the comments raised by all three reviewers and I feel the manuscript is now substantially improved. It now provides a much stronger experimental and clearer case for their conclusions.

Comments for the author

All reviewers comments have now been addressed.

Reviewer 3

Advance summary and potential significance to field

© 2023. Published by The Company of Biologists under the terms of the Creative Commons Attribution License (https://creativecommons.org/licenses/by/4.0/).

This is the first study describing scRNAseq data on Sertoli cells ablated for the key sex-determining factor NR5A1. The data hightlight the effects of NR5A1 ablation at specific time points on cellular plasticity

The data also provide data to support the hypothesis that that PTM cells and adult LC derive from a single population of Wnt5a+ steroidogenic progenitors

Comments for the author

The authors have considerably improved the English style and structure of the manuscript taking into account the comments of all of the reviewers.
The comment on the NR5A1 AMH-cre construct --- Excision should be due AMH expression – a direct target of NR5A1 at a time that is later than 12.5 in SC. The authors state that the excision is occurring between 12.5 and 13.5 because of their observations. They cite effects of genetic background which may well explain this effect.
The authors have addressed the comments on DMRT1 and the results of NR5A1 ablation at specific points. It is much clearer now for the reader esp the supplemental Table S5.
In the discussion line 328 & 330 the authors refer to Developmental Sex Disorders (DSD). I would remove this abbreviation as it will lead to confusion with the established abbreviation human Disorders/Differences of Sex Development (DSD).

Editor's note:

Please consider revising the abbreviation "DSD" (reviewer 3) in the page proof of the paper.

© 2023. Published by The Company of Biologists under the terms of the Creative Commons Attribution License (https://creativecommons.org/licenses/by/4.0/).
